# Impact of cross-linking stoichiometry on the structure and allergenicity of glutaraldehyde-polymerized allergen extracts

**DOI:** 10.3389/fimmu.2026.1748277

**Published:** 2026-02-26

**Authors:** Sandra Sivill, Marcos Viñuela, Diego García-Puentes, Lucia Moreno-Serna, Emilio Nuñez-Borque, Rodrigo Jiménez-Saiz, Maria Vila-Gonzalo, Miguel Fernández-Arquero, Irene Real-Arévalo, Salvador Iborra, Jose Luis Subiza, Jose Fernando Cantillo

**Affiliations:** 1 Research and Development Department, Inmunotek, S.L., Alcalá de Henares, Madrid, Spain; 2 Servicio de Inmunología, Hospital Clínico San Carlos, Madrid, Spain; 3 Fundación Investigación, Hospital Clínico San Carlos, Madrid, Spain; 4 Department of Immunology, Instituto de Investigación Sanitaria Hospital Universitario de La Princesa (IIS-Princesa), Universidad Autónoma de Madrid (UAM), Madrid, Spain; 5 Faculty of Experimental Sciences, Universidad Francisco de Vitoria (UFV), Madrid, Spain; 6 Department of Medicine, McMaster Immunology Research Centre (MIRC), Schroeder Allergy and Immunology Research Institute (SAIRI), McMaster University, Hamilton, ON, Canada

**Keywords:** allergens, glutaraldehyde-modified extracts, hypoallergenicity, immunotherapy, polymerization

## Abstract

**Introduction:**

Polymerized allergen extracts are widely used in allergen immunotherapy (AIT) to reduce allergenicity and enhance safety. However, the impact of cross-linking stoichiometry on the structural and allergenic properties of these polymers remains insufficiently characterized. This study systematically investigates how varying glutaraldehyde-to-protein ratios influence the resulting polymers and their potential relevance for AIT.

**Methods:**

Pollen extracts from *Phleum pratense* and *Betula verrucosa* were polymerized using five different glutaraldehyde-to-protein ratios. The resulting polymers were structurally characterized using SDS-PAGE, nuclear magnetic resonance (NMR), transmission electron microscopy (TEM), and mass spectrometry (MS). Allergenicity was evaluated by measuring the immunoglobulin E (IgE) reactivity through enzyme-linked immunosorbent assay (ELISA), Western blotting, and mast cell activation test (MAT). Immunogenicity was assessed by analyzing the serum-specific IgG and *ex vivo* lymphocyte responses in mice immunized with the least allergenic polymer formulation.

**Results:**

All cross-linking conditions produced polymers with distinct differences in size, morphology, and yield. Despite retaining similar peptide profiles to native allergens, as confirmed by MS, the polymers exhibited increased stability, as shown by NMR, and significantly reduced allergenicity, according to ELISA and MAT. Notably, increased polymer size and density, as determined by NMR and TEM, correlated with lower allergenicity. The most extensively cross-linked high-density polymers, optimized for minimal allergenicity, elicited immunogenic responses comparable to those induced by native extracts when tested against unmodified allergens.

**Conclusion:**

Cross-linking stoichiometry critically shapes the structural and immunological properties of polymerized allergen extracts. Adjusting the glutaraldehyde-to-protein ratio to produce highly polymerized, dense polymers enables a well-balanced profile of safety and efficacy for use in AIT.

## Graphical Abstract

**Figure:**
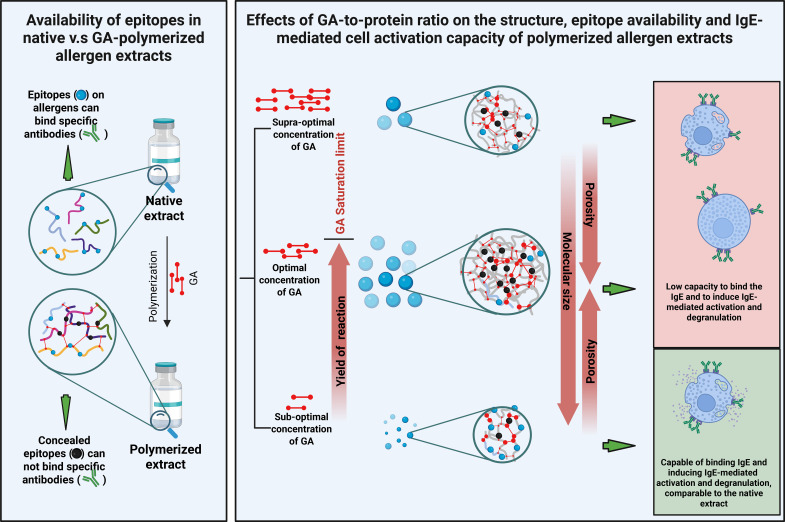

## 1 Introduction

Allergen immunotherapy (AIT) is the only disease-modifying treatment for immunoglobulin E (IgE)-mediated allergic diseases and aims to induce long-term immune tolerance. Nevertheless, the risk of allergen-induced adverse reactions, in particular during the dose escalation (buildup) phase, continues to limit wider adoption. To improve safety while preserving efficacy, chemically modified allergens (allergoids) were introduced in the 1970s as hypoallergenic preparations (1). In Europe, allergoid-based AIT is now routinely used because it improves tolerability, reduces systemic reactions, and shortens the otherwise prolonged buildup phase required for native extracts (NEs).

Glutaraldehyde (GA) is the cross-linking reagent most commonly employed by European manufacturers to produce hypoallergenic allergoids (2, 3). As a bifunctional aldehyde, GA reacts with ε-amino groups of lysine residues, forming Schiff bases (imines) that drive extensive intermolecular cross-linking and polymerization.

Despite the clinical success of GA-modified extracts in AIT (4–13), the molecular determinants of their hypoallergenicity remains poorly defined. Early work by Patterson and colleagues, relying on antibody-based assays and basic chromatographic methods, provided only partial structural information on GA-cross-linked extracts (2, 14–16). Modern high-resolution techniques [e.g., advanced mass spectrometry (MS), nuclear magnetic resonance (NMR), or cryo-electron microscopy] have not yet been systematically applied to connect the polymer architecture to the residual allergenicity. Limited evidence suggests two mechanisms, not mutually exclusive, by which GA polymerization reduces IgE binding: i) physical concealment of epitopes within the polymer matrix (2) and ii) chemical alteration or destruction of those epitopes (17, 18). If epitope shielding is a principal mechanism, the extent of cross-linking is likely critical. Model protein studies have shown that the GA concentration is positively correlated with the cross-link density, the polymer size, and the reaction yield, but inversely with porosity (19–21). Whether these relationships hold for complex allergen extracts and how they translate into reduced allergenicity remain unclear.

Here, we systematically varied the GA-to-protein ratios to generate polymerized extracts (PEs) from two clinically important pollen sources: *Phleum pratense* (Pp) and *Betula verrucosa* (Bv). We characterized the resulting polymer morphology, the cross-link density, and the degree of epitope masking and correlated these parameters with IgE binding and effector cell activation. Our goal was to identify structural correlates of hypoallergenicity that can guide the rational design of safer, yet immunogenically competent, AIT formulations.

## 2 Materials and methods

### 2.1 Native and polymerized extracts and source of purified allergens

Defatted pollen of *P. pratense* and *B. verrucosa* (Iber-Polen, Jaén, Spain) were extracted as previously described for *P. pratense* (22). Briefly, pollen was stirred in phosphate-buffered saline (PBS; pH 7.4, 4°C), clarified by centrifugation (15,000 × *g*, 10 min, 4°C), subjected to tangential flow ultrafiltration (cutoff pore size, 100 kDa), dialyzed against water, sterile-filtered (0.22 µm), and lyophilized. Freeze-dried aliquots were stored at −80°C and served as NEs (Pp-N and Bv-N for *P. pratense* and *B. verrucosa* NEs, respectively).

NEs were reconstituted at 2 mg protein/ml in 0.1 M phosphate buffer (pH 7.4). The protein content prior to polymerization was determined with the Bradford assay (Bio-Rad, Hercules, CA, USA). Cross-linking reactions were performed at a final volume of 20 ml by adding GA and PBS to reach five protein-to-GA ratios, i.e., 0.1×, 0.2×, 1×, 5×, and 10×, relative to the standard reference condition of 25 mM GA (designated as P1). The resulting preparations—P0.1 (2.5 mM GA), P0.2 (5 mM GA), P1 (25 mM GA), P5 (125 mM GA), and P10 (250 mM GA)—were incubated at 22°C for 3 h under stirring. The reactions were quenched with glycine using 125 mM for the standard reference condition (P1, 25 mM GA) and adjusting the glycine concentration proportionally for all other GA concentrations. Samples were sterile-filtered (0.22 µm), and excess of GA, non-polymerized allergens, and other residual substances were removed with five cycles of ultrafiltration using Vivaspin 20 protein concentrator devices (100 kDa cutoff; Sartorius, Göttingen, Germany) through buffer exchange from PBS to Milli-Q water to guarantee conductivity values below 1,000 µS/cm. The flow-through obtained after the first ultrafiltration step, corresponding to the low-molecular-weight fraction (LMWF), was retained for analysis, while the retained high-molecular-weight fraction (HMWF) was considered the PEs. The protein content of both fractions was determined with the Bradford assay. The polymerization efficiency (**Table 1**) was estimated from the protein concentration in the LMWFs. As the initial protein content in the extracts is known, the fraction of protein remaining in the LMWFs was used to infer the amount retained in the HMWF (PEs).

**Table 1:** Overall analysis of polymers (P) prepared from *Phleum pratense* (Pp) and *Betula verrucosa* (Bv) extracts.

Allergen	Preparation	Protein concentration in the LMWFs (µg/mL)	% of protein retained in the LMWFs	% of protein retained in the HMWF PEs (efficiency)	Transmission electron microscopy (TEM)		sIgE by ELISA (media from 5 individual sera)	Biological potency (BPIC_50_)^†^	Strength of IgE binding (avidity index)	MAT		
					Mean size (µm2)	Porosity (mean intensity)				% of CD63^+^ cells	% of CD107a^+^ cells	% of β-hexosaminidase activity
*P. pratense*	Pp-N	–	–		–	–	1.5440	0.198	1.087	3.978	4.498	7.180
	Pp-P10	930	46.5	53.5	0.2112	146.1	0.5042	364.900	1.076	1.094	1.292	3.280
	Pp-P5	533	26.6	73.4	0.2032	125.6	0.5547	144.600	0.933	1.138	1.274	3.700
	Pp-P1	71.0	03.5	96.5	0.6453	183.7	0.6327	204.300	0.941	1.052	1.240	3.460
	Pp-P0.2	55.0	02.7	97.3	0.2230	147.2	1.0070	12.200	0.800	1.654	1.934	4.540
	Pp-P0.1	66.0	03.3	96.7	0.1505	155.0	1.3600	1.700	0.819	2.716	3.092	5.820
*B. verrucosa*	Bv-N	–	–		–	–	1.3660	0.067	0.690	9.606	9.210	9.520
	Bv-P10	1902	95.1	04.9	1.9990	165.1	0.2813	0.759	0.824	2.398	1.978	2.340
	Bv-P5	695.0	34.8	65.2	1.4180	167.8	0.3844	1.315	0.888	2.422	1.940	2.540
	Bv-P1	316.0	15.8	84.2	2.9210	181.6	0.5564	23.580	0.801	3.148	2.742	2.960
	Bv-P0.2	327.0	16.4	83.6	1.2730	130.3	1.0740	58.130	0.773	6.478	6.206	6.680
	Bv-P0.1	603.0	30.2	69.8	0.6551	140.0	0.9469	112.100	0.725	8.126	7.908	7.200

*LMWFs*, low-molecular-weight fractions; *HMWF*, high-molecular-weight fraction; *PEs*, polymerized extracts; *sIgE*, specific immunoglobulin E.

^†^µg of preparation necessary to inhibit 50% of the IgE reactivity against the native extract.

PEs were sterile-filtered, lyophilized, and stored at −80°C until used. The final Pp-N- and Bv-N-derived samples were designated as Pp-P10, Pp-P5, Pp-P1, Pp-P0.2, and Pp-P0.1 and as Bv-P10, Bv-P5, Bv-P1, Bv-P0.2, and Bv-P0.1, respectively.

Native Bet v1 and Phl p5 were purified as previously described (23) and served as monospecific controls.

### 2.2 SDS-PAGE, Western blot, and MS

Of the NEs or PEs, 5 μg was mixed with loading buffer and boiled for 5 min. The sample was separated in Any kD™ Mini-PROTEAN^®^ TGX™ Precast Protein Gelsat (Bio-Rad) or 4%–20% Mini-PROTEAN^®^ TGX™ Precast Protein Gels, at 280 V, using a Mini-PROTEAN II apparatus (Bio-Rad). Precision Plus Protein All Blue Standards (Bio-Rad) was used as the molecular weight (MW) reference. Visualization of the separated molecules was achieved by staining with colloidal Coomassie (GelCode Blue Stain Reagent; Life Technologies, Carlsbad, CA, USA). Gels were documented using a ChemiDoc Imaging System (Bio-Rad) with the “Protein gels–Coomassie stain” preset and subsequent color rendering in Image Lab 6.1 software to show the Coomassie blue staining.

For Western blot (WB), proteins in the polyacrylamide gel were transferred onto a 0.45-µm nitrocellulose membrane using a Transblot Semi-Dry Electrophoresis transfer cell (Bio-Rad) at 20 V for 30 min. The membranes were blocked with PBS containing 0.25% Tween-20 (PBS-T) containing 5% bovine serum albumin (BSA) for 60 min. The membranes were incubated with sera from individuals sensitized to *P. pratense* or *B. verrucosa* or 1:5,000 diluted sera from mice immunized with Phl p 5, Bet v 1, Pp-N, or Bv-N. For the IgE binding studies, five pooled sera (1:50) from subjects sensitized to Pp-N (AbBaltis, Sittingbourne, UK) and Bv-N (PlasmaLab, Everett, WA, USA) were used. For murine IgG reactivity, the membranes were incubated with mixtures of two sera from mice immunized with Phl p 5, Bet v 1, Pp-N, or Bv-N diluted 1:10,000. After incubation, the membranes were incubated with 1:2,000 horseradish peroxidase (HRP)-conjugated mouse anti-human IgE (SouthernBiotech, Birmingham, AL, USA) or 1:2,000 HRP-labeled anti-mouse IgG (Sigma-Aldrich, St. Louis, MO, USA). Protein–antibody binding was detected with an ECL Prime detection reagent (GE Healthcare Life Sciences, Little Chalfont, UK) in a ChemiDoc imaging system (Bio-Rad). Precision Plus Protein WesternC Standards (Bio-Rad) was used as the MW marker, previously incubated with Precision Protein StrepTactin–HRP Conjugate (Bio-Rad) for detection.

The protein and allergen composition were determined by MS (24), conducted at the Proteomics Unit, National Center for Biotechnology (CNB-CSIC, Madrid, Spain). Samples were resuspended in 500–1,000 µl of 2.5% (*w*/*v*) SDS, 25 mM triethylammonium bicarbonate (TEAB), 5 mM Tris(2-carboxyethyl)phosphine hydrochloride (TCEP), and 10 mM chloroacetamide (CAA). Lysates were incubated at 60°C for 30 min to reduce and alkylate protein cysteine residues. The protein extracts were centrifuged at 18,000 × *g* for 10 min, and the supernatant was transferred into a new tube and quantified using Pierce™ 660 nm Protein Assay Reagent supplemented with Ionic Detergent Compatibility Reagent according to the manufacturer’s instructions. For automatic SP3-based protein digestion, the samples were processed with the Opentrons OT-2 robot in the presence of MagReSyn^®^ HILIC microparticles (ReSyn Biosciences, Edenvale, South Africa) and digested at 37°C overnight using trypsin (1:33 enzyme/protein ratio) and Lys-C (1:500 enzyme/protein ratio) enzymes. The eluted peptides were dried in a speed vacuum and quantified by fluorimetry (QuBit, Paris, France) according to the manufacturer’s instructions.

For nano-liquid chromatography coupled to electrospray ionization tandem mass spectrometry (nanoLC-ESI-MS/MS) analysis, 500 ng of each sample was individually analyzed using an UltiMate 3000 Nano HPLC System (Thermo Fisher Scientific, Waltham, MA, USA) coupled online to an Orbitrap Exploris™ 240 mass spectrometer (Thermo Fisher Scientific). Each sample [500 ng in 5 µl of sample resuspended in mobile phase A: 0.1% formic acid (FA)] was loaded on a 50-cm × 75-μm µPAC Neo C18 analytical column at 50°C and separated at a flow rate of 250 nl/min using a 120-min gradient ranging from 2% to 95% mobile phase B [80% acetonitrile (ACN) in 0.1% FA]. Data acquisition was performed using a data-dependent top 20 method, in full scan positive mode, scanning 375–1,200 *m*/*z*. MS1 scans were acquired at an Orbitrap resolution of 60,000 at *m*/*z* 200, with normalized automatic gain control (AGC) target of 300%, a radio frequency (RF) lens of 80%, and an automatic maximum injection time (IT). The 20 most intense ions from each MS1 scan were selected and fragmented with a higher-energy collisional dissociation (HCD) of 34%. The resolution for the HCD spectra was set to 45,000 at *m*/*z* 200, with a normalized AGC target of 50% and an automatic maximum IT. Isolation of precursors was performed with an isolation window of 0.7 *m*/*z* and 45 s of exclusion duration. Precursor ions with charge states from 2^+^ to 5^+^ were included.

Raw instrument files were processed using Proteome Discoverer (PD) version 3.1.1.93 (Thermo Fisher Scientific). MS2 spectra were searched using Mascot Server v2.8.1 (Matrix Science, London, UK) against the Poeae or Betulaceae UniProtKB databases containing the most common laboratory contaminants (cRAP database with 70 sequences). All searches were configured with dynamic modifications for pyrrolidone from Q (−17.027 Da) and oxidation of methionine residues (+15.9949 Da) and static modification as carbamidomethyl (+57.021 Da) on cysteine, monoisotopic masses, and trypsin cleavage (maximum of two missed cleavages). The peptide precursor mass tolerance was 10 ppm, and the MS/MS tolerance was 0.02 Da. The false discovery rate (FDR) for proteins, peptides, and peptide spectral matches (PSMs) was kept at 1%.

Precursor ion quantitation was also performed in Proteome Discoverer using the “Minora” feature in the processing method and the “Feature Mapper” and “Precursor Ions Quantifier” nodes in the consensus step. Protein abundance was calculated by summing the sample abundance rates of the connected peptide groups (using unique+razor peptides).

### 2.3 Nuclear magnetic resonance

One-dimensional ^1^H spectra and a pseudo-two-dimensional (2D) diffusion-ordered spectroscopy (DOSY) were acquired in a Bruker Avance NEO 400-MHz spectrometer using standard pulse sequences included in TopSpin 4.1.1 software to analyze the physicochemical properties, the molecular size, and the stability of the native and polymerized preparations following standard protocols from our laboratories.

Briefly, the ^1^H spectra were recorded by applying a zg30 pulse sequence, which presents a 30° excitation pulse instead of the conventional zg program (90° excitation pulse). Samples were prepared in D_2_O, and experiments were acquired at 298 K on a 400-MHz Bruker Avance NEO spectrometer. Data were processed with MestReNova 15.0.1 (Mestrelab, Santiago de Compostela, Spain) and TopSpin 4.1.1 (Bruker, Madrid, Spain) software. These experiments were acquired with 64 scans, a spectral width (SW) of 14 ppm, and a free induction decay (FID) size of 65k data points. The DOSY spectra were obtained using the standard pulse sequence, acquiring 32 gradient points, with 16 scans each, between 2% and 98% gradient intensity using a diffusion time delay of big delta (Δ) 0.30 s and little delta (*δ*) 2,000 μs to achieve a wide pulse gradient. Data were analyzed using the pseudo-2D spectra and also obtaining the sum projection in the diffusion dimension of each one. Successful polymerization was inferred from line broadening, chemical shift dispersion, and decreased diffusion coefficients. Extract stability was assessed over 3 months at 4°C by monitoring changes in the integrated signal intensities relative to the baseline spectrum (25).

For the estimation of MWs, a calibration protocol using DOSY experiments was performed as previously described (26), with protein standards at 5 mg/ml in D_2_O: ovalbumin (44 kDa), conalbumin (75 kDa), thyroglobulin (669 kDa), and Blue Dextran (2,000 kDa), all from Bio-Rad. Proteins were registered following the pulse sequence *ledpbgp2s* (Bruker). Due to the heterogeneous nature of the extract, eight signals with varying diffusion coefficient values were selected for calculation to reach a closer approximation of the molecular size distribution for each sample. The corresponding log*D* values were extrapolated from the calibration curve and were used to estimate the MW of each component in the preparation.

### 2.4 Transmission electron microscopy

Experiments were carried out at the Spanish National Centre for Electron Microscopy (ICTS) at the Complutense University, Madrid, Spain. Samples (1,000 µg protein/ml) were adsorbed onto carboncoated copper grids, negatively stained with 2 % (w/v) uranyl acetate, and examined on a JEOL JEM-1010 TEM operated at 80 kV and fitted with a Gatan Erlangshen ES500W CCD camera (Gatan, Pleasanton, CA, USA). Apparent porosity was estimated from micrographs using ImageJ by segmenting the particle areas and calculating the mean gray value distributions. Darker, highdensity regions were interpreted as thicker, less porous domains.

### 2.5 Dynamic light scattering

Experiments were carried out at the Molecular Interactions Facility at the CIB Margarita Salas. Measurements were performed using a Protein Solutions DynaPro MS/X instrument (Protein Solutions, Ankeny, IA, USA) at 20°C and a 90° light scattering cuvette. Data were collected and analyzed with the Dynamics v6 software. Particle size distributions are reported as intensity-weighted hydrodynamic radii.

We thank the staff of CIB Margarita Salas Molecular Interactions Facility for technical assistance with the dynamic light scattering (DLS) assays.

### 2.6 ELISA and competition experiments

Detection of human specific IgE against native or PEs was performed with ELISA using the five commercial sera mentioned above.

All sera were tested in titration experiments to determine the dilution to reach optical densities (ODs) of ~1.5 when tested against the NEs. The NEs and PEs were tested at the defined dilution. Briefly, microplates were coated overnight at 4°C with 10 μg/ml of the antigens in 0.05 M carbonate–bicarbonate buffer, pH 9.4. After three washes with PBS-T, nonspecific reactivity was blocked by 1 h of incubation with PBS-T containing 1% BSA. Wells were washed again and incubated overnight with diluted sera at 4°C. Subsequently, the wells were washed again and incubated for 1 h with 1:2,000 HRP-conjugated mouse anti-human IgE (SouthernBiotech). After a final washing step, 3 mg/ml of the peroxidase substrate *o*-phenylenediamine dihydrochloride (OPD) (Sigma-Aldrich) dissolved in citrate buffer (0.1 M citrate buffer with 0.03% H_2_O_2_, pH 5.5) was added to the wells to allow detection of the reaction. The reaction was stopped by adding 10% HCl and read at 492 nm in a spectrophotometer (Synergy Mx, BioTek, Winooski, VT, USA).

Competitive ELISA was performed to analyze the capacity of the preparations to block the IgE reactivity against the NEs and expressed as biological potency (BP; micrograms of competitor necessary to block 50% of the reaction). ELISA experiments were performed as described; however, serial dilutions of the inhibitor (NE or PE; 0.2–0.0004 µg/ml) were pre-incubated with pooled serum (1:60) for 2 h at 37°C and transferred to the coated wells. After completion of all steps, the inhibition percentage was calculated using the following equation: percent inhibition = 100 − [(OD of serum with competitor/OD of serum without competitor) × 100]. Sera adsorbed with PBS-T containing 1% BSA without competitor served as a positive control.

### 2.7 Avidity analysis by potassium thiocyanate elution ELISA

To measure the strength of the binding between PEs and the IgE, a potassium thiocyanate (KSCN) ELISA-elution assay was performed (27). The experimental procedure was similar to the ELISA for the IgE binding determination, with three differences: 1) the serum pool was added to the microplates at a dilution to reach ODs of ~1.5; 2) the incubation with sera lasted 2 h; and 3) after incubation with sera, KSCN diluted in PBS-T containing 1% BSA was added to the plates for 30 min at room temperature. The KSCN molar concentrations were: 2.8, 2.5, 2.2, 1.9, 1.6, 1.3, 1, 0.7, 0.4, and 0.1. The reaction was revealed as described above.

The results were expressed as percentage of reduction and avidity index, defined as the KSCN concentration required to elute 50% of sample–IgE complexes. To determine the avidity index, we calculated the reduction of absorbance after KSCN treatment relative to the samples without treatment.

### 2.8 Mastocyte activation test and β-hexosaminidase assay

Tests were performed using the mast cell line LAD2, kindly provided by Drs. Metcalfe and Kirshenbaum (National Institute of Allergy and Infectious Diseases, Bethesda, MD, USA) (28). LAD2 cells were cultured in complete StemPro-34 media (Gibco, Waltham, MA, USA) at 37°C and 5% of CO_2_, as described (29). In addition, to increase the expression of the high-affinity IgE receptor (FcϵRI) before any experiment, the cells were cultured for 5 days with 10 ng/ml of recombinant human interleukin-4 (IL-4; R&D Systems, Minneapolis, MN, USA) before any experiments.

LAD2 cells were sensitized as reported (29). In brief, 10^6^ cells/ml were incubated overnight with a pool of sera from five patients allergic to *B. verrucosa* or *P. pratense* (1:25). Subsequently, the cells were centrifuged at 265 × *g* for 5 min and resuspended in fresh media. LAD2 cells were then challenged for 10 min in a 96-well flat-bottomed plate (0.5 × 10^6^ cells/ml) with increasing doses (0.1, 0.5, 1, 5, 25, and 50 μg/ml) of native or PEs of *B. verrucosa* and *P. pratense*. On the other hand, for the analysis of individual patients, sensitization was performed with serum from each subject (1:25) and the cells were activated with both extracts at doses of 5 and 10 μg/ml. After stimulation, flow cytometry analysis was performed as described (30). The cells were blocked with human TruStain FcX (1:50; BioLegend, San Diego, CA, USA) in ice-cold FACS buffer (2.5 mM EDTA and 0.5% BSA in PBS) for 15 min. The cells were stained for 30 min with a mix of fluorochrome-conjugated antibodies/FITC–FcεRI (AER-37; 1:100), PE–CD117 (104D2; 1:600), BV421–CD63 (H5C6; 1:200), APC–CD107a (H4A3; 1:100), and PE–cyanine7–IgE (MHE-18; 1:100), all from BioLegend. Moreover, cell viability was assessed with eFluor780 dye (1:2,000; Thermo Fisher Scientific). Finally, the cells were washed with FACS buffer and analyzed in a FACSCanto II flow cytometer (Becton Dickinson, Franklin Lakes, NJ, USA). On average, 10,000 events of live and single cells were recorded. Analysis was performed with the FlowJo v10 program. Non-stimulated and polyclonal α-human IgE-stimulated cells (Sigma-Aldrich) were used as negative and positive activation controls, respectively. A representative gating strategy for a positive sample is shown in **Supplementary Figure 1**, outlining the sequential gating steps applied for the flow cytometry analysis.

The activity of β-hexosaminidase was measured as previously reported, with minor modifications (29). In brief, 40 µl of the supernatant from each condition was placed in a 96-well flat-bottomed plate, in duplicate, and 40 µl of the β-hexosaminidase substrate solution (2 mM *p*-nitrophenyl *N*-acetyl β-d-glucosamine diluted in 100 mM citrate buffer) was added. The culture medium alone and the supernatants from Triton-lysed cells were included to determine the absorbance background and to measure the total levels of β-hexosaminidase, respectively. Subsequently, the plate was incubated for 45 min at 37°C in the dark and 80 µl of 1 M NaOH was added afterward to stop the reaction. Finally, the absorbance (OD) was measured at 405 nm using the GloMax Discover (Promega, Madison, WI, USA) microplate reader. The percentage of β-hexosaminidase activity was calculated according to the following formula:


Degranulation (%) = [(OD supernatant − OD background)/(OD total lysis − OD background)] × 100

Degranulation (%) = [(OD supernatant − OD background)/(OD total lysis − OD background)] × 100

### 2.9 Immunization and lymphoproliferative response

BALB/cByJ mice were obtained from Charles River Laboratories (France). All animal experiments were conducted at the Experimental Medicine and Surgery Department, Hospital Clínico San Carlos (Madrid, Spain), in accordance with the ARRIVE guidelines and following the European Directive 2010/63/EU on the protection of animals used for scientific purposes. National regulations were also followed, specifically Spanish R.D. 53/2013. All procedures were approved by the Institutional Animal Care and Ethics Committee.

Immunization of mice was performed to examine the immunogenicity and the humoral responses induced by the Pp-P1 and Bv-P1 preparations and Pp-N and Bv-N. Balb/c mice (groups of five animals) were subcutaneously immunized three times every 2 weeks with a 1:1 mixture of 100 µg of NE or PEs (Pp-N, Bv-N, Pp-P1 and Bv-P1) and aluminum hydroxide (Croda, Snaith, UK) adjuvant–PBS per dose. Immunization with an equivalent volume of adjuvant solution was used as a control. At 7 days after the final dose, the blood and spleens were collected for subsequent assays. Specific murine antibodies against NEs were detected using ELISA as described above, with minor modifications. Antigen coating and blocking were performed as described above. For total IgG, IgG1, and IgG2a, mouse sera were diluted 1:2,000. For specific IgE detection, mouse sera were diluted 1:4. Detection of specific antibodies was achieved using 1:2,000 HRP-labeled antibodies (anti-mouse IgG, IgG1, IgG2a, or IgE from Sigma-Aldrich) and OPD substrate as explained.

The IL-10 measurement followed the methodology previously described by our group (31). Spleens were isolated aseptically, minced, and filtered through sterile 100-µm steel filters. Erythrocyte-free cells suspended in PBS containing 0.5% fetal bovine serum (FBS; Gibco) were stained with 2 μM carboxyfluorescein succinimidyl ester (CFSE) for 10 min in the dark. Afterward, the cells were washed with PBS containing 2% FBS. CFSE-stained cells were cultured into 48-well plates at a concentration of 2 × 10^6^ cells/well in the presence of 5 or 50 µg/ml Pp-N or Bv-N extract. Cells cultured with 2.5 μg/ml of concanavalin A (Sigma-Aldrich) were used as a positive proliferation control. Cells without stimulant were used as the negative control. After 5 days, proliferation was measured on an FC-500 flow cytometer (Beckman Coulter, Brea, CA, USA). IL-10 was measured in 48-h culture supernatants using a specific ELISA quantification kit (R&D Systems). Data were analyzed using the FlowJo v.10 software and GraphPad Prism software.

### 2.10 Statistical analysis

Data were analyzed with GraphPad Prism 10 software. For the analysis of MAT, comparisons among conditions were performed using two-way ANOVA followed by Dunnet’s test. Data were considered significant at *p* < 0.05.

For the analysis of the antibody levels in immunized mice, lymphoproliferation, and IL-10 measurements, comparisons for non-parametric data were performed using the Kruskal–Wallis test, while that for parametric data was performed using one-way ANOVA.

For exploration and visualization of the correlation between all variables examined in the physicochemical, molecular, and biological behavior of polymers, we used principal component analysis (PCA) to statistically aggregate these variables, reducing the number of observed variables into a smaller number of principal components (PCs). For correct aggregation of the variables, the data from Pp-N-derived samples were normalized relative to the highest value in each group, and the same was done for the Bv-N-derived samples. PCs (PC1 and PC2) were selected with the largest eigenvalues that together explained 90% of the total variance. The results are displayed in a PC1 *versus* PC2 graph.

## 3 Results

### 3.1 Characterization of *P. pratense* and *B. verrucosa* extracts polymerized at different glutaraldehyde ratios

Five PEs were generated from each NE by decreasing (0.1× and 0.2×) or increasing (5× and 10×) the GA concentration relative to the reference condition (1× = 25 mM, P1). The resulting preparations are denoted Pp P10 → Pp P0.1 and Bv P10 → Bv P0.1 for *P. pratense* and *B. verrucosa*, respectively.

**Figure 1:**
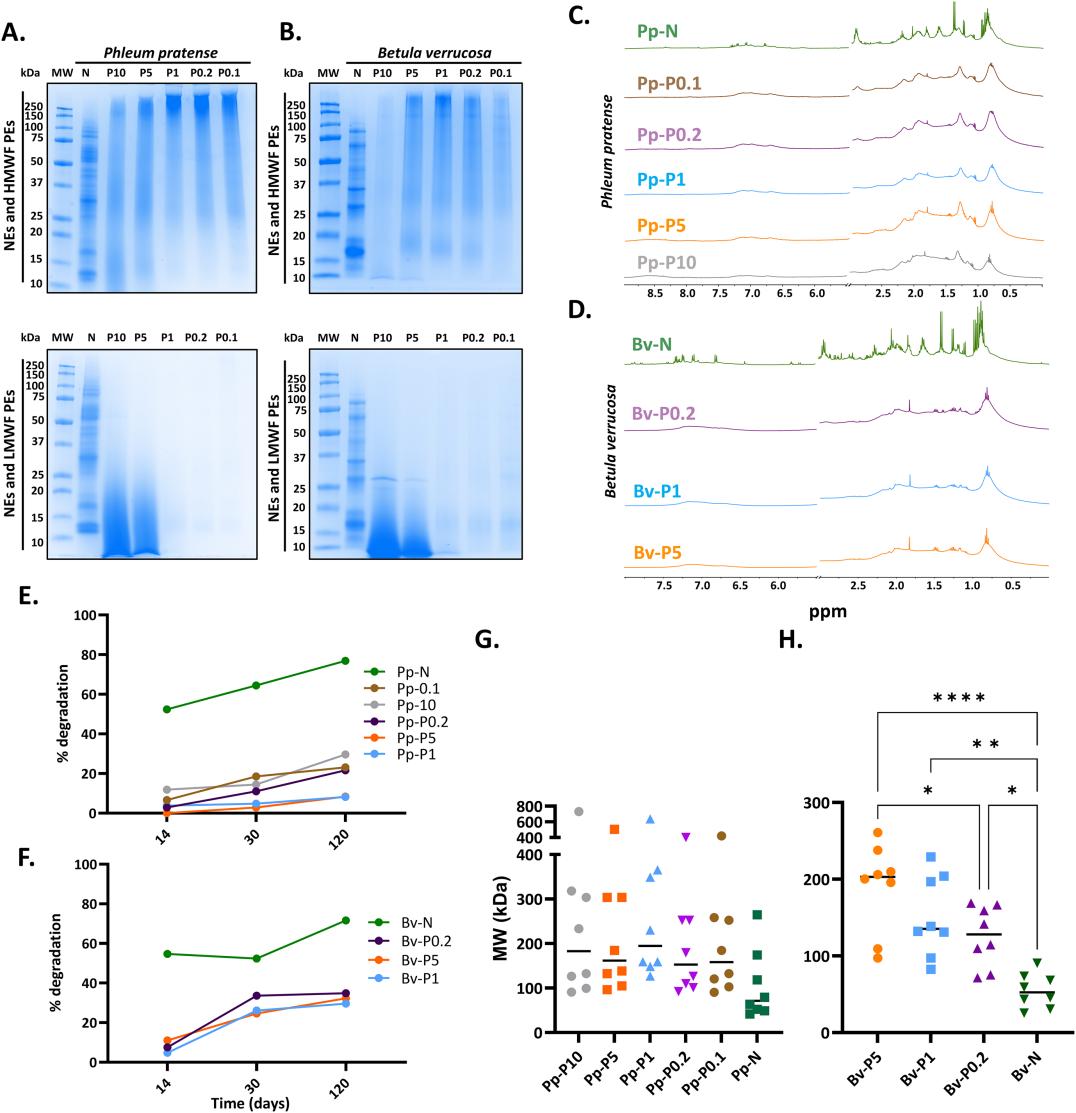
Electrophoretic profiling of native (NE) and polymerized extracts (PEs) (high-molecular-weight fractions, HMWFs), as well as low-molecular-weight fractions (LMWFs), and nuclear magnetic resonance studies. **(A, B)** SDS-PAGE analysis of the NEs and PEs from *Phleum pratense***(A)** and *Betula verrucosa***(B)**. For each sample, the HMWF PEs (*top*) and LMWFs (*bottom*) are shown. **(C, D)**^1^H-NMR spectra were recorded for the NE and PE Pp-N **(C)** and Bv-N **(D)** samples. **(E, F)** Degree of degradation for NEs and PEs from *P. pratense***(E)** and *B. verrucosa***(F)** after comparing the ^1^H-NMR spectral signal intensities at multiple time points. **(G, H)** Molecular weight distribution in descending order of the NEs and PEs from *P. pratense***(G)** and *B. verrucosa***(H)** calculated from the calibration curves, as described in **Supplementary Figure 3**. When a statistically significant difference in the size distribution was found, the *p*-value is indicated at the *top of the graphic*. *p < 0.05, **p < 0.01, ****p < 0.001. Two samples, i.e., Bv-P10 and Bv-P0.1, were not analyzed due to their limited availability resulting from the low polymerization yield. *MW*, molecular weight marker; *N*, native extract.

The polymerization efficiencies, which were calculated from the residual LMWFs (**Table 1**), ranged from 5% (P10) to 97% (P1). SDS-PAGE analysis confirmed successful polymerization, with all PEs displaying a predominant band above 250 kDa, while the NEs showed multiple bands from 10 to 250 kDa (**Figures 1A, B**, top). A smear appeared only in the LMWF of P10 and P5 (**Figures 1A, B**, bottom) and in the glycine–GA control reactions (**Supplementary Figure 2**), suggesting the presence of glycine–GA adducts.

One-dimensional ^1^H NMR spectroscopy further supported the formation of HMWF PEs, as evidenced by line broadening and the signal attenuation in all PEs compared with their respective NEs (**Figures 1C, D**), consistent with extensive cross-linking. The polymer stability over time was assessed through longitudinal NMR measurements. By comparing the spectral signal intensities at multiple time points (T0 → T3), degradation was monitored as described (25). NEs showed progressive signal loss indicative of degradation, whereas PEs remained largely stable. Notably, Pp-P1 and Bv-P1 showed the highest stability over the 4-month period (**Figures 1E, F**).

**Figure 2:**
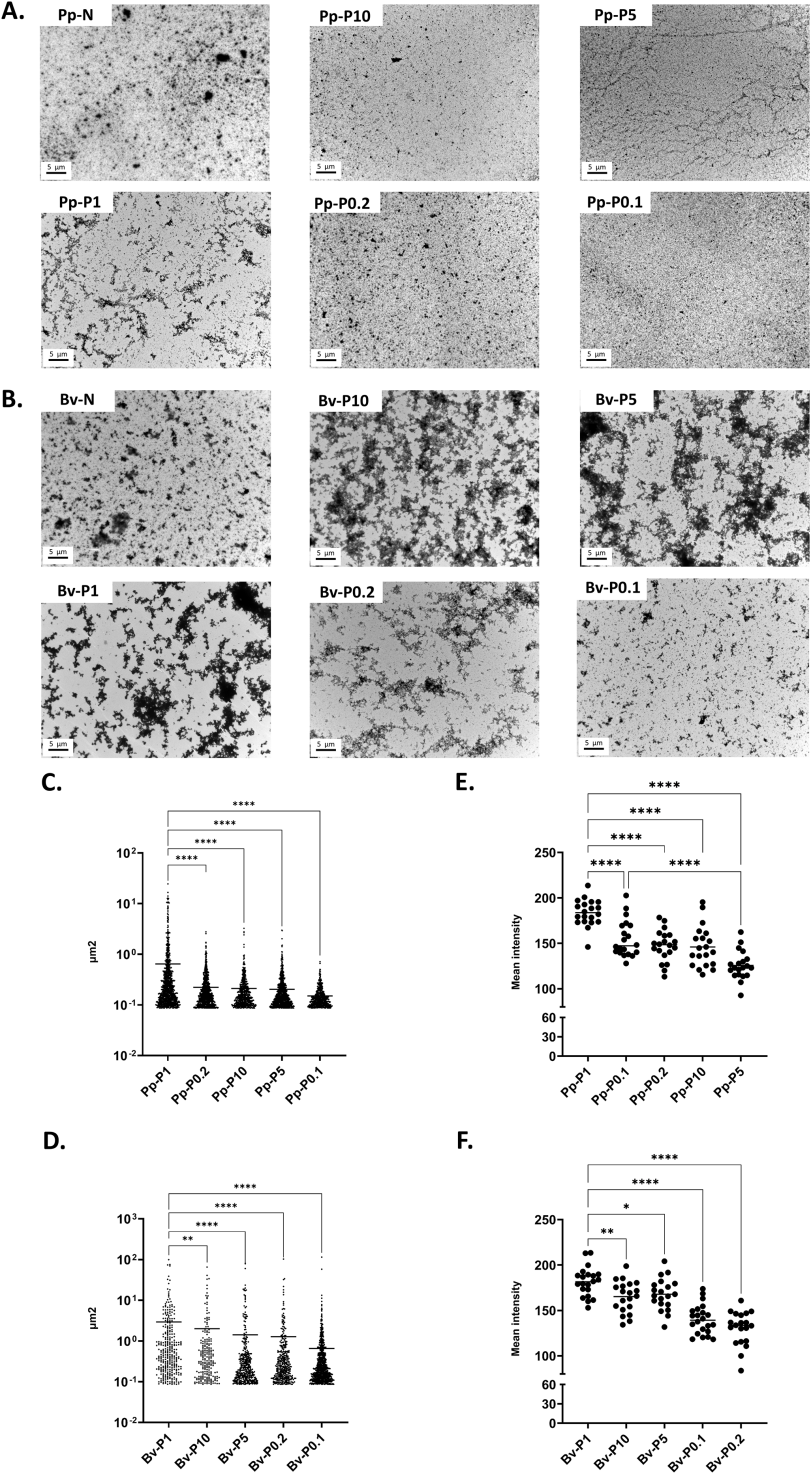
Visualization of native and polymerized Pp-N and Bv-N and estimation of their mean particle size and porosity. **(A, B)** Images of native (*N*) and polymerized (*P*) extracts from *Phleum pratense***(A)** and *Betula verrucosa***(B)** obtained at the same magnification (×8,000) and protein concentration (2,500 µg/ml). Representative images (labeled in the *top left corner of each figure*) are shown. **(C–F)** Estimation of the average size and porosity inferred from the images taken from polymers derived from Pp-N **(C, E)** and Bv-N **(D, F)**. The *p*-values for samples that presented significant statistical differences after multiple comparisons are displayed at the *top of the graphic*. *p < 0.05, **p < 0.01, ****p < 0.001.

The hydrodynamic size distribution of the polymers was examined using two orthogonal approaches: DOSY-NMR and TEM. The apparent MW distribution derived from DOSY (**Supplementary Figure 3**) ranged from 100 to 700 kDa for *P. pratense* PEs (**Figure 1G**) and from 90 to 300 kDa for *B. verrucosa* PEs (**Figure 1H**). In both cases, P1 extracts exhibited the largest average molecular sizes. These findings were corroborated by TEM, which showed that P1 samples formed the largest structures, while extreme low GA conditions (0.1×) led to smaller, fragmented morphologies (**Figures 2A–D**). In addition, we attempted to validate these results using DLS on Bv-N-derived PEs (**Supplementary Figure 4**). DLS resolved only four to six broad populations with high polydispersity indices, each likely encompassing multiple particle sizes, suggesting that this technique does not have sufficient resolution to accurately resolve the high degree of polydispersity present in our samples. DLS has limited resolution toward large species with hydrodynamic radii approaching or exceeding the micrometer range, which further restricts the reliable characterization of these samples by this technique.

Morphological and porosity differences were evident in the TEM analysis. While NEs appeared as irregular and dispersed particles, possibly representing protein aggregates, PEs prepared at moderate to high GA ratios (e.g., Pp-P1, Pp-P5, Bv-P1, and Bv-P5) formed dense or filamentous networks. In contrast, low and high GA concentrations (e.g., Pp-P0.1 and Pp-P10) produced smaller, less interconnected structures (**Figures 2A, B**). Image-based porosity analysis revealed the following increasing order of porosity (**Figures 2E, F**):

*P. pratense*: Pp P1 > Pp P0.1 > Pp P0.2 > Pp P10 > Pp P5
*B. verrucosa*: Bv P1 > Bv P10 > Bv P5 > Bv P0.1 > Bv P0.2

Together, these results suggest that GA stoichiometry modulates the key structural parameters of PEs, including the yield, size, morphology, porosity, and stability.

### 3.2 Protein and allergen composition of glutaraldehyde-polymerized extracts at different cross-linking stoichiometries

To determine which allergens remained detectable following GA polymerization, the NEs and PEs were analyzed using LC-MS/MS and immunoblotting.

**Figure 3:**
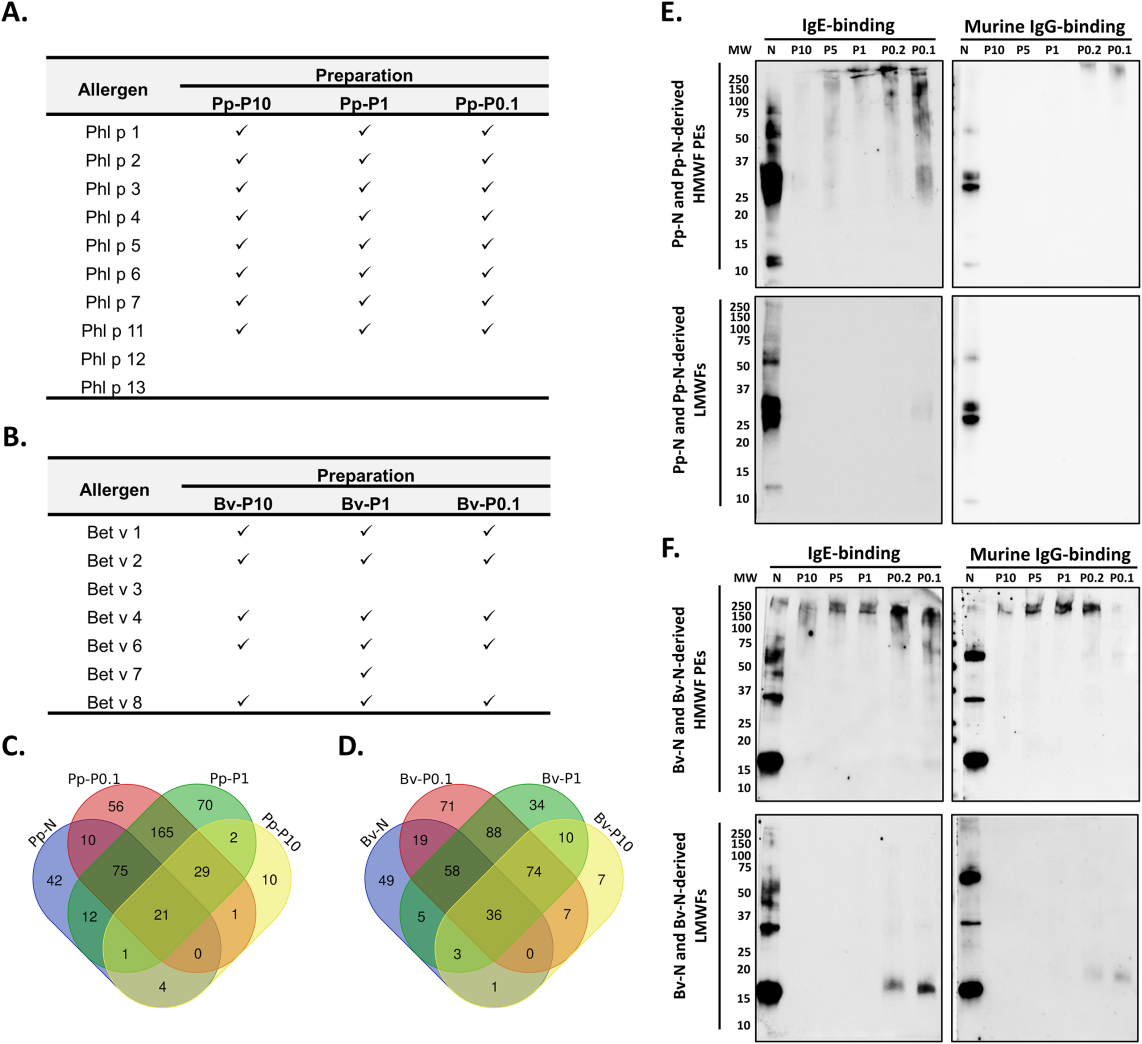
Allergen and protein content in native and polymerized Pp-N and Bv-N. Mass spectrometry (MS) and Western blotting (WB) were used for the analysis. MS was performed on native extract (NE) and on samples from three representative polymerization conditions: P10, P1, and P0.1. **(A, B)** Allergen content determined by MS for each NE and polymerized extract (PE) summarized for *Phleum pratense***(A)** and *Betula verrucosa***(B)**. Allergens correspond to the official allergenic proteins from *P. pratense* and *B. verrucosa* at the date (https://www.allergen.org/). **(C, D)** Venn diagrams showing the concurrence of proteins identified by MS in Pp-N and Bv-N and the representative PEs. Common elements among samples are indicated, and their protein and allergen content are indicated in **Supplementary Table 1**, **S2**. **(E, F)** Pp-N- and Bv-N-derived high-molecular-weight fraction (HMWF) PEs previously resolved by SDS-PAGE were tested for IgE (*left panel*) and murine IgG binding (*right panel*) using WB. Antibody reactivity was tested for IgE reactivity with pooled sera from sensitized individuals and for IgG reactivity using sera from a mouse immunized with the corresponding native extract. SDS-PAGE and WB were performed in parallel to analyze 10 µl of low-molecular-weight fractions (LMWFs) [*lower panel* in **(E, F)**]. In this case, differences in the intensity of the signals are partially dependent on the differences of the amount of material contained in the analyzed volume. *MW*, molecular weight marker; *N*, native extract.

Proteomic profiling by LC-MS/MS was performed on three representative polymerization conditions from each pollen source (P10, P1, and P0.1) (**Figures 3A, B**). For *P. pratense*, all examined PEs (Pp-P10, Pp-P1, and Pp-P0.1) contained the major allergens, apart from Phl p 12 and Phl p 13. In contrast, allergen recovery from *B. verrucosa* was more variable: only Bv-P1 retained the full panel of known allergens, except for Bet v3, which was absent from all PEs. In addition, several proteins were detected uniquely in the PEs, but not in their corresponding NEs (**Figures 3C, D**), suggesting altered detectability of the high-molecular-weight species following cross-linking. A summary of these proteins is provided in **Supplementary Table 1** for the Pp-derived samples and in **Supplementary Table 2** for the Bv-derived samples, while the corresponding raw datasets are available in *Supplementary data 1*–*4* (for Pp) and *Supplementary data 5*–*8* (for Bv).

WB analysis corroborated the MS findings and provided additional insights into IgE and IgG epitope availability. In *P. pratense* (**Figure 3E**), residual IgE binding at >250 kDa was observed across all PEs. However, polyclonal IgG from mice immunized with Pp-N (**Figure 3E**) or Phl p 5 (**Supplementary Figure 5A**) only lightly cross-linked the Pp-P0.2 and Pp-P0.1 samples, implying that stronger cross-linking (≥P1) leads to epitope masking or modification. Importantly, no IgG reactivity was detected in the LMWFs, confirming effective polymerization (**Figure 3E**; **Supplementary Figure 5A**).

For *B. verrucosa*, IgE and murine IgG reactivity specific for Bv-N and Bet v 1 were consistently detected at >250 kDa in all polymerized samples. Notably, Bv-P10 exhibited low IgE and IgG signal intensities, whereas Bv-P0.1 showed strong IgE but weak IgG binding (**Figure 3F**; **Supplementary Figure 5B**). The intermediate conditions (Bv-P5, Bv-P1, and Bv-P0.2) displayed similar binding profiles for both antibody types.

In some LMWFs, discrete immunoreactive bands persisted: a 28-kDa IgE binding band in Pp-P0.1 and a 17-kDa band in Bv-P0.2 and Bv-P0.1 (**Figures 3E, F**; **Supplementary Figure 5B**), likely representing unpolymerized monomeric allergens that escaped cross-linking.

Taken together, these results show that GA polymerization retains the majority of the clinically relevant allergens.

### 3.3 Cross-linking stoichiometry governs IgE binding and mast cell activation

To evaluate the effect of GA cross-linking stoichiometry on allergenicity, we assessed the IgE binding and effector cell activation using a series of established immunoassays.

**Figure 4:**
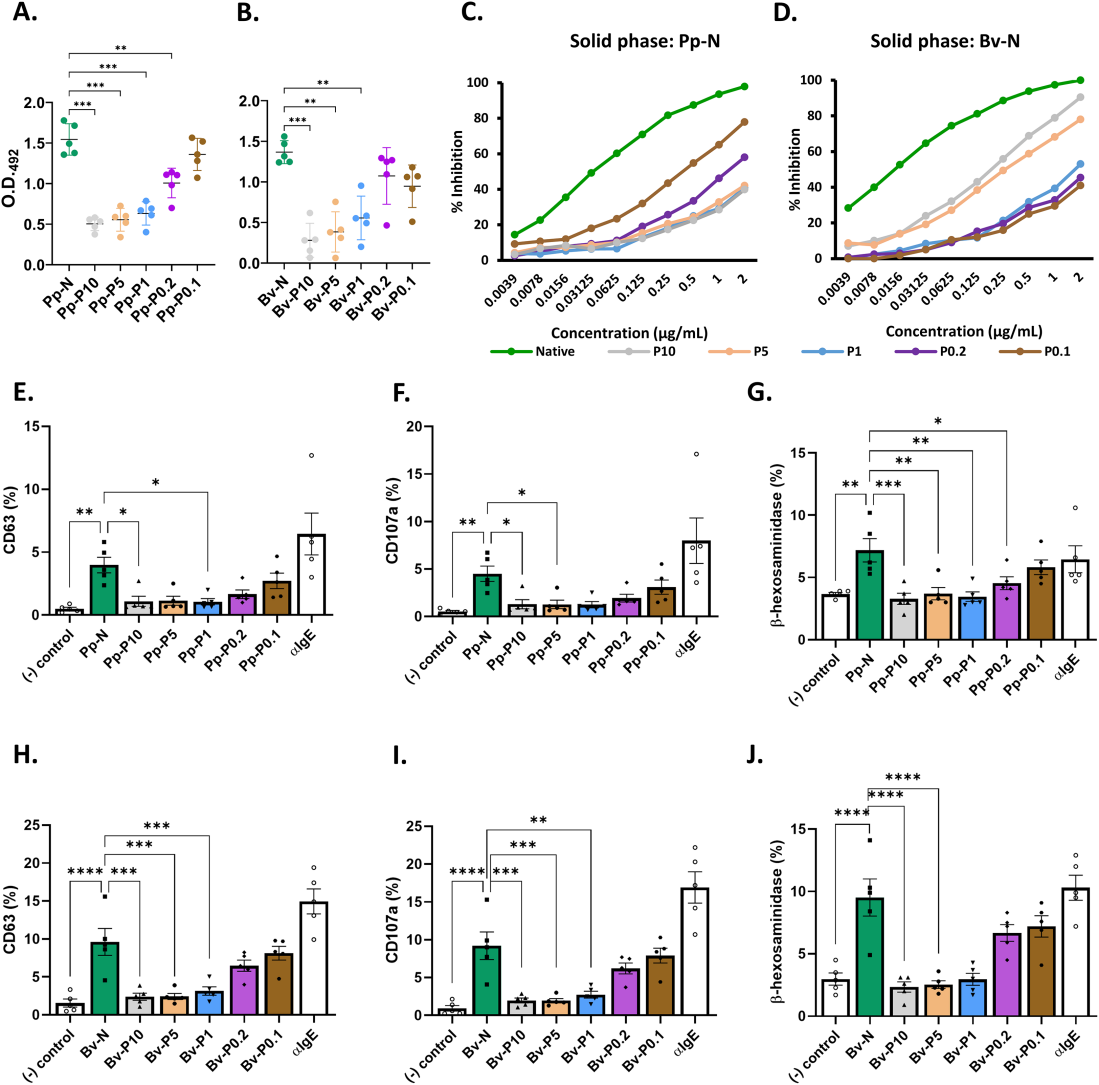
Allergenicity and IgE-mediated activation of native and polymerized Pp- and Bv-N-derived preparations. **(A–D)** Allergenicity was tested using indirect ELISA **(A, B)** and competitive ELISA **(C, D)** for *Phleum pratense*- and *Betula verrucosa*-derived samples, respectively. IgE-mediated activation of cells was studied in mast cell activation test (MAT) experiments. **(E–J)** Mast cells previously sensitized with individual sera from subjects with IgE sensitization to each allergenic source (*n* = 5) were analyzed for the expression of the activation markers CD63, CD107a, and β-hexosaminidase activity upon stimulation with native extract (NE) and polymerized extract (PE) Pp-N **(E–G)** and Bv-N **(H–J)**. Negative and positive activation controls correspond to non-stimulated cells and cells stimulated with polyclonal α-human IgE, respectively. When statistically significant differences in the specific IgE (ELISA) or in the MAT experiments were found, the *p*-value is indicated at the *top of the graphic*. *p < 0.05, **p < 0.01, ***p < 0.001, ****p < 0.001.

All PEs displayed reduced IgE binding capacity compared with their respective NEs, as determined with indirect ELISA (**Figures 4A, B**). This reduction was statistically significant for all preparations, except those produced with the lowest GA concentrations (i.e., Pp-P0.1, Bv-P0.1, and Bv-P0.2).

Consistent with these findings, the ELISA inhibition assays showed a stoichiometry-dependent reduction in BP. At a concentration of 2 µg/ml, Pp-N achieved 97.8% inhibition of IgE binding (BP = 0.198), while the most extensively cross-linked Pp-P10 only inhibited 39.8% (BP = 364.9) (**Figure 4C** and **Table 1**). A similar trend was observed for *B. verrucosa*, with Bv-N showing complete inhibition (BP = 0.067) and Bv-P0.1 exhibiting only 41% inhibition (BP = 112.1) (**Figure 4D** and **Table 1**), indicating a diminished capacity of the PEs to block IgE recognition of NEs.

Although the overall KSCN elution profiles were comparable across extracts (**Supplementary Figure 6**), the avidity indices, used here to estimate the strength of IgE–epitope interactions, showed measurable differences (**Table 1**). For *P. pratense*, the index declined from 1.087 in Pp-N to 0.800 in Pp-P0.2; for *B. verrucosa*, it dropped from 0.960 in Bv-N to 0.725 in Bv-P0.1. These changes reflect a weakening of the IgE binding strength associated with specific GA-to-protein ratios.

The functional consequences of these differences were evaluated by MAT. The lowest activation levels (expression of the CD63 and CD107a surface markers and β-hexosaminidase release) were observed for the most extensively cross-linked P10, P5, and P1 formulations, both for *P. pratense*-derived (**Figures 4E–G**; **Supplementary Figures 7A, C–E**) and *B. verrucosa*-derived PEs (**Figures 4H–J**; **Supplementary Figures 7B, F–H**). In contrast, P0.2 and P0.1 induced mast cell responses comparable to those elicited by the NEs.

These results suggest that GA-mediated polymerization reduces both IgE binding and effector cell activation in a stoichiometry-dependent manner.

### 3.4 Multivariate relationships among structural and biological variables

**Figure 5:**
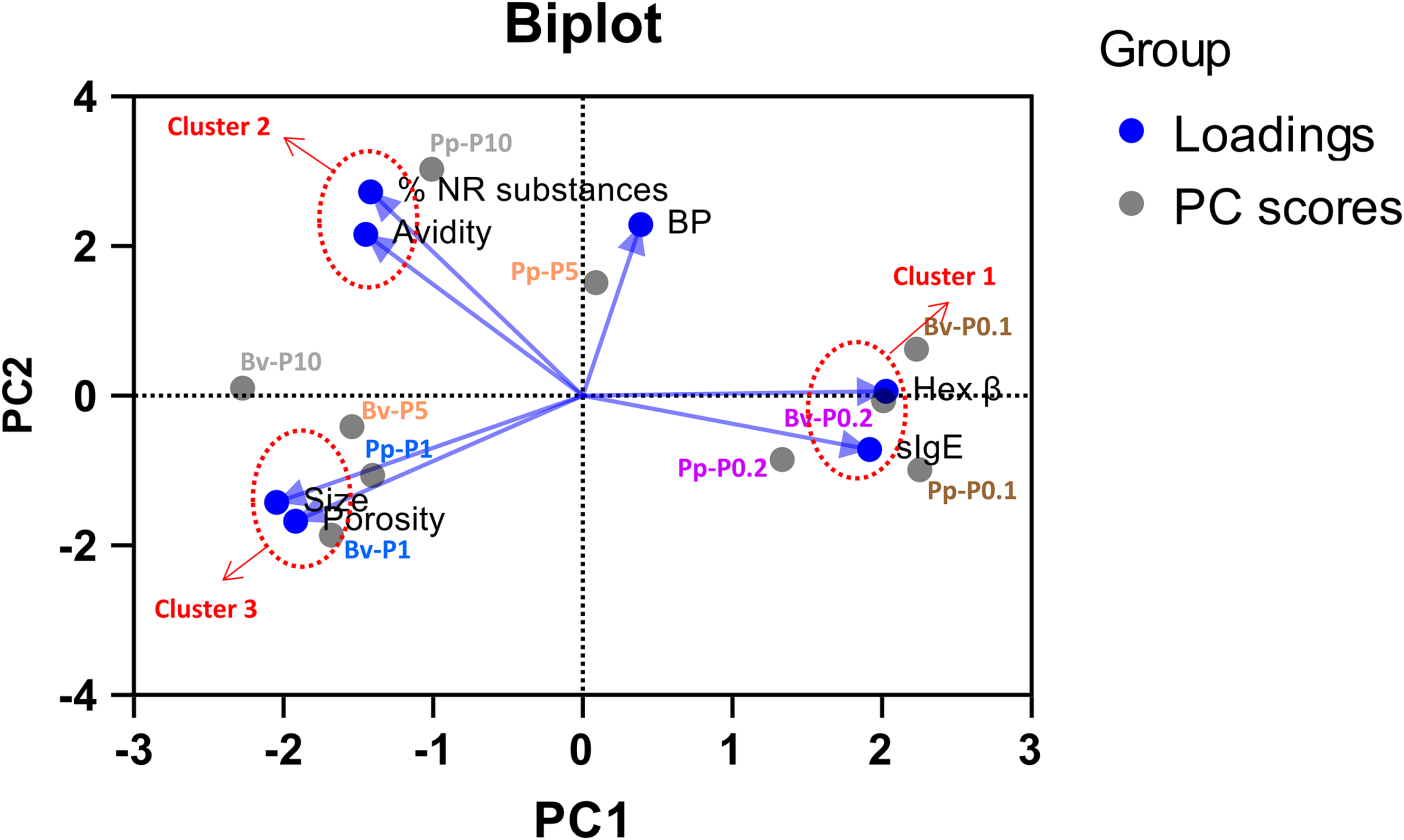
Results of the principal component analysis based on seven variables. The data obtained from the analysis of native Pp-N and Bv-N and their corresponding polymerized extracts (PEs) were normalized at each individual variable and analyzed in conjunction. Screen biplot of principal component 1 (PC1) *versus* PC2: PC1 and PC2 represent the first and second PCs, accounting for the highest and the second highest proportion of variance in the dataset, respectively. Loadings that correspond to the tested variables are plotted and shown in *blue*. The PC scores, each of them corresponding to different PEs, are shown in *gray*. Three main clusters of loadings (clusters 1–3) are indicated in the *dashed red circle*. The variables considered in the analysis were: specific IgE, β-hexosaminidase activity, size, porosity, avidity index, biological potency, and percentage of non-reactive substances.

To better understand how structural and immunological properties are interrelated across the different polymerization conditions, PCA was performed using all measured physicochemical, molecular, and biological parameters (see *Section 3.2*). The resulting biplot (**Figure 5**) revealed three distinct clusters that corresponded to specific GA cross-linking stoichiometries.

The first cluster was defined by high mast cell activation and elevated IgE binding and included samples generated at the lowest GA-to-protein ratios (Pp-P0.1, Bv-P0.1, and Pp-P0.2). This group reflected the least effective cross-linking, associated with poor allergen masking and high effector cell activation.

The second cluster comprised samples with high IgE binding, avidity, and low polymerization yield. It was associated with the highest GA concentrations (Pp-P10 and Bv-P10). These results suggest that, while excessive GA results in dense structures with strong IgE–epitope interactions, it may also limit the efficiency of polymer formation.

The third cluster included preparations with large sizes, high density, and reduced allergenicity and was centered around the reference GA-to-protein ratios (Pp-P1 and Bv-P1). These samples consistently exhibited the most favorable structural and immunological profiles, supporting their selection as optimal formulations for further immunological evaluation.

Interestingly, BP, as assessed using ELISA inhibition, did not cluster with any of the other variables. This indicates that BP may be influenced by additional factors not fully captured by the parameters included in this analysis.

### 3.5 Immunogenicity of the least allergenic polymers (P1)

To evaluate whether the most hypoallergenic polymers (P1) retained immunogenicity, BALB/c mice were immunized with P1 PEs or their corresponding NEs.

**Figure 6:**
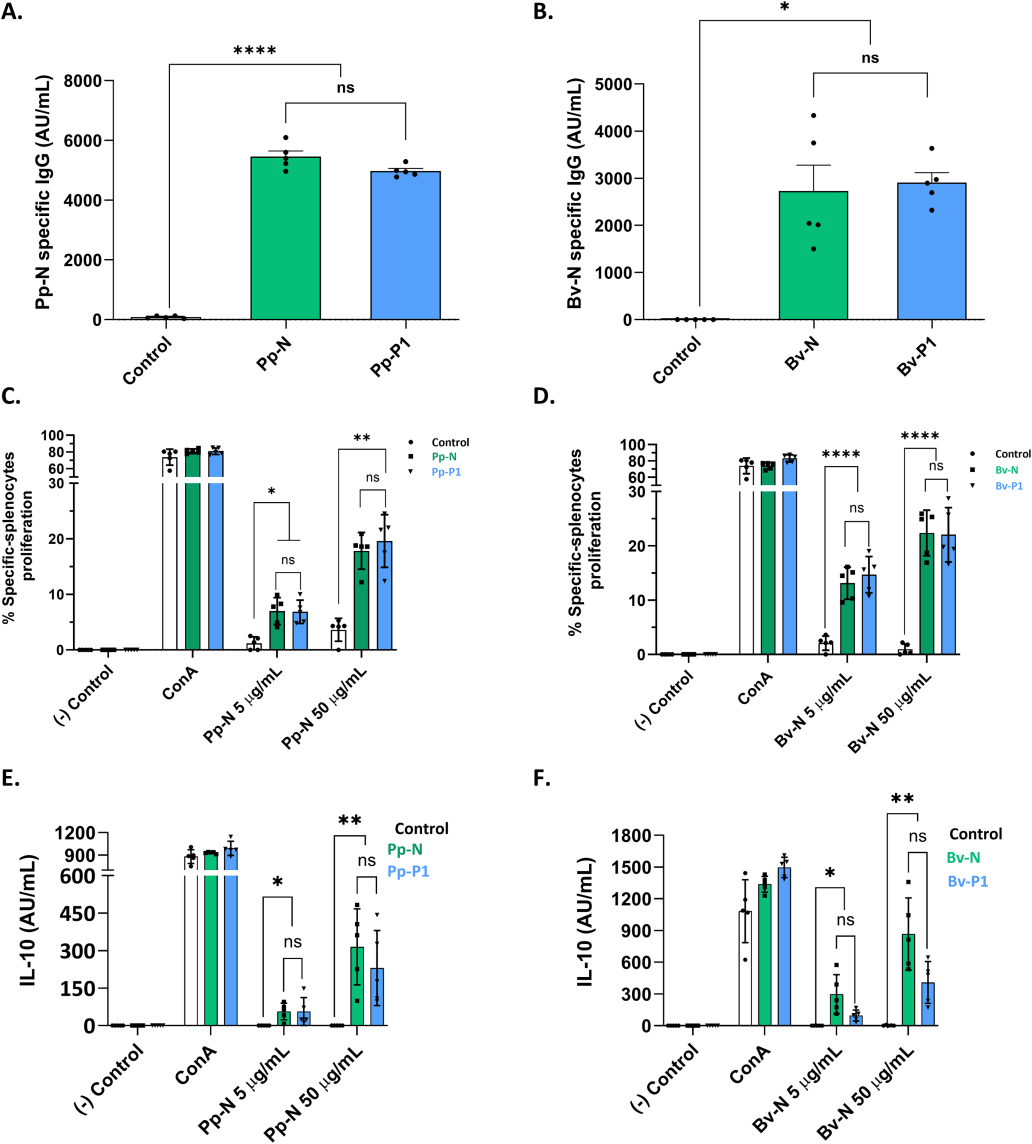
Serum antibody, *ex vivo* lymphoproliferation response, and IL-10 production in mice immunized with native extracts (NEs) and P1 polymerized extracts (PEs). **(A, B)** Specific IgG against Pp-N or Bv-N in the serum of mice immunized with NEs and P1-PEs from *Phleum pratense* and *Betula verrucosa*, respectively, was detected by ELISA. **(C–F)** Lymphoproliferative cell response **(C, D)** and supernatant IL-10 levels **(E, F)** in spleens from mice after intraperitoneal immunization with Pp-N and Pp-P1 **(C, E)** and Bv-N and Bv-P1 **(D, F)** stimulated with 5 and 50 μg/ml of the corresponding NE. When a statistically significant difference was found, the *p*-value is indicated at the *top of the graphic*. *p < 0.05, **p < 0.01, ****p < 0.001.

Both PEs and NEs from *P. pratense* and *B. verrucosa* elicited comparable levels of allergen-specific IgG (**Figures 6A, B**), including the IgG1 and IgG2a isotypes (**Supplementary Figures 8A, B, E, F**), indicating preserved humoral responses. All groups showed detectable IgE titers, with no statistically significant differences between the PEs and NEs (**Supplementary Figures 8C, G**). Notably, Bv-P1 induced specific IgE levels that were indistinguishable from the adjuvant-only control. Evaluation of the IgG2a/IgE ratio revealed similar values for Bv-N and Bv-P1, while Pp-P1 showed a slightly lower, but not statistically significant, ratio compared with Pp-N (**Supplementary Figures 8D, H**).

Cellular immune responses were also maintained. Splenocytes from P1-immunized mice showed proliferation (**Figures 6C, D**) and IL-10 production (**Figures 6E, F**) upon stimulation with NEs at levels comparable to those observed in NE-immunized animals, but significantly higher than the negative control, supporting the preservation of T-cell reactivity.

Taken together, these results demonstrate that P1 polymers, despite their reduced allergenicity and high structural density, retain the immunogenic profile of NEs.

## 4 Discussion

This study systematically dissects how GA cross-linking stoichiometry shapes the structure and allergenic behavior of PEs from two clinically relevant allergen sources: grass (*P. pratense*) and birch (*B. verrucosa*). Varying the GA-to-protein ratio modulated epitope accessibility and, consequently, the IgE binding and effector cell activation. Despite few studies investigating the structural complexity of PEs, conventional hydrodynamic methods such as DLS or size exclusion chromatography (SEC) capture only a portion of the heterogeneity inherent to PEs (32). By integrating i) DOSY, ii) TEM, iii) LC-MS/MS, and iv) antibody-based assays, we obtained a multi-scale portrait of the polymer architecture and the epitope presentation. Some proteins, including allergens, may be detected in the PEs, but not in the NEs. A possible explanation is that differential reactivity allows certain proteins to cross-link more efficiently, potentially due to the accessibility of reactive residues, leading to their relative enrichment within the polymer network and enhanced detectability. Although absolute MW estimates from DOSY depend on imperfect calibration standards, the technique remains a sensitive, accessible means of comparing the relative size distributions. TEM complements NMR by directly visualizing the network morphology and porosity. Unlike DLS or SEC, whose resolving power is limited for highly polydisperse and non-spherical polymeric systems, the combined use of DOSY and TEM enables relative size comparisons while directly capturing the broad size distribution, irregular morphology, and filamentous architectures characteristic of PEs.

The stoichiometric nature of GA cross-linking is inherently complex, with variable outcomes dependent on the reaction conditions (33), particularly the GA-to-protein ratio (19–21). Our results revealed that the reaction yield varies with GA concentration, reaching its highest level under our standard P1 GA-to-protein condition. At higher GA concentrations, saturation appears to inhibit cross-linking, likely due to the accumulation of free aldehyde. Because GA exists in multiple reactive forms in aqueous solution (34), excess GA can undergo secondary reactions and is preferentially scavenged by glycine during quenching, leading to heterogeneous reaction products, manifesting as a diffuse smear on SDS-PAGE rather than discrete bands. Although these products were not directly characterized, this interpretation is consistent with the known chemistry of GA and the observed electrophoretic behavior. While no direct correlation between the GA concentration and the PE size or porosity was observed, the significant variation in polymer size aligns with prior findings (35). However, the NMR and TEM results indicate that the PEs prepared at the P1 GA-to-protein ratio are among the densest and largest, although the absolute dimensions differed between the grass- and birch-derived preparations, underscoring source-specific factors.

In addition to influencing the size and porosity, variations in the GA concentration may have several effects on epitopes, affecting their availability and antibody reactivity (18) and consequently altering the IgE-mediated effector activation (18, 36) and the overall allergenicity (37). In our study, all PEs showed reduced IgE binding capacity, with none exhibiting IgE binding similar to or higher than the NE, as shown for some allergoids (38), but rather changes in epitope accessibility and binding strength. High GA-to-protein ratio preparations (P5–P10) showed both low IgE binding and inhibitory potency; however, the optimal extent of cross-linking was extract-dependent, as evidenced by the source-specific differences in avidity (high for Pp-P10 and low for Bv-P10). MAT followed a bell-shaped relationship: maximal attenuation occurred at P1 or above, whereas under-cross-linked P0.1–P0.2 polymers retained appreciable activity, consistent with other observations (16, 39). This indicates that a stronger reduction in biological activity is achieved at a specific size range, while smaller sizes result in a less pronounced reduction.

The PCA clustered variables into three domains: i) high allergenicity/effector activation (low GA-to-protein ratios); ii) high avidity, but poor yield (high GA-to-protein ratios); and iii) large, dense, hypoallergenic polymers (P1). BP segregated independently, implying additional determinants beyond the measured variables. This likely reflects intrinsic characteristics of IgE–allergen interactions, such as valency and affinity, which can vary substantially between extracts and thus contribute to differences in BP independently of the polymer morphology or the GA-to-protein ratio. Our proposed model (Graphical abstract) suggests that, at the optimal P1 ratio, the reaction yield is maximized, producing highly polymerized, larger, and denser preparations with reduced allergenicity and IgE-mediated activation. This likely reflects limited antibody access to epitopes due to 1) larger polymers that may physically conceal more epitopes within the matrix and 2) increased density that restricts antibody penetration. Importantly, these effects appear independent of the antibody binding strength, as functional group addition does not significantly reduce the binding avidity. Even if the IgE epitopes retain reactivity, the reduced exposure of epitopes limits their interaction with IgE, preventing full activation of effector cells. On the other hand, smaller and less dense PEs retain higher allergenicity and IgE-mediated activation. This supports the hypothesis that epitope concealment rather than epitope modification drives allergenicity reduction. Nevertheless, we cannot fully exclude a contribution of epitope modification. For instance, lysine residues that are key reactive sites under physiological conditions may be chemically occupied during GA cross-linking, potentially altering epitope surfaces and affecting allergen recognition. In addition, densely arranged epitopes on the polymer surface likely reduce the interaction with sparsely distributed IgE on effector cells, minimizing mast cell activation, similar to the model proposed for allergen-displaying virus-like particles (40). A less likely but plausible scenario is that polymerization induces conformational changes that alter the epitope spectrum. As IgE binding is dependent on the native 3D structure of the allergen (41), such alterations could reduce IgE recognition. In contrast, IgG binding, which is more reliant on linear epitopes, is less impacted, potentially enhancing the blocking capacity and engagement of the inhibitory FcγRIIb receptor (42, 43). Regardless of the mechanism, all scenarios suggest that the polymer arrangement is key in determining allergenicity and effector cell activation.

A recurrent concern with allergoids is that reduced allergenicity may come at the cost of immunogenicity (38, 44). Encouragingly, the P1 polymers, those with the lowest mast cell activation, elicited specific IgG, IgG1, IgE, and IgG2a antibodies, IgG2a/IgE antibody ratio, splenocyte proliferation, and IL-10 production indistinguishable from NEs. Thus, the structural rearrangements that curtail IgE engagement do not impede T- or B-cell priming, satisfying a key requirement for effective AIT (45, 46). Nevertheless, we acknowledge that safety assessment would be strengthened by validation in allergic or sensitized mouse models.

In conclusion, modulating GA cross-linking stoichiometry generates a continuum of polymer sizes, densities, and porosities, which translates into predictable changes in allergenicity. The reference condition (P1) yields highly polymerized, dense aggregates that effectively reduce IgE binding and effector cell activation while preserving immunogenicity, positioning it as an optimal formulation for clinical application.

## Data Availability

The original contributions presented in the study are included in the article/**Supplementary Material**. Further inquiries can be directed to the corresponding author.

## Supplementary material

Supplementary Figure 1 Flow cytometry gating strategy for LAD2 cells used in mast cell activation assays. Cells were first identified based on forward scatter (FSC) and side scatter (SSC) properties to exclude debris, followed by singlet discrimination to remove doublets. Viable cells were selected by exclusion of the viability dye eFluor™ 780. CD117⁺ mast cells with high surface expression of FcεRI were then gated. Mast cell activation was defined by surface expression of the degranulation markers CD63 and CD107a within the viable singlet CD117⁺FcεRI^high^ population. Positive indicates sensitized mast cells stimulated with native extract, whereas negative represents unstimulated cells.

Supplementary Figure 2 Electrophoretic Analysis of LMWFs and bimolecular GA-Glycine Reaction products. Equal amounts of LMWFs (normalized) derived from the different polymerization of Pp-N **(A)** and Bv-N **(B)** were resolved by SDS-PAGE. To elucidate the nature of the <100 kDa intense smear that appeared in the LMWFs from P10 and P5 PEs, bimolecular GA–Glycine reaction resembling the conditions used for PEs preparation were resolved by SDS-PAGE, normalized by volume **(C)** or by the amount of GA-glycine **(D)**, that were loaded in the wells. MW: Molecular weight marker; N: Native extract

Supplementary Figure 3 Calibration curve and estimation of NEs and PEs molecular weights distribution by NMR data. **(A)** Plot of logD versus logMW for 4 globular proteins: ovalbumin (44 kDa), conalbumin (75 kDa), ferritin (669 kDa), and dextran (2,000 kDa). Superimposed 2D-DOSY spectra of Pp-N **(B)** and Bv-N **(C)** derived polymers. Colour codes are as follows: green for native extracts, gray for P10, orange for P5, blue for P1, magenta for P0.2, and brown for P0.1. **(D and E)** Column-wise sum projection of the DOSY spectra for the Pp-N and Bv-N derived polymers, respectively.

Supplementary Figure 4 Dynamic Light Scattering. Hydrodynamic radius vs Intensity distribution plots (right) and Intensity-weighted DLS peak parameters (left) are shown for Bv-P10 **(A)**, Bv-P5 **(B)**, Bv-P1 **(C)**, Bv-P0.2 **(D)** and Bv-P0.1 **(E)**.

Supplementary Figure 5 Complementary WB analysis of NEs, HMWFs PEs, and LMWFs. **(A)** Pp-N and its derived polymers (top), and corresponding non-reactive fractions (bottom), were tested for murine anti-Phl p 5 IgG. **(B)** Bv-N and its derived polymers (top), and non-reactive (<100 kDa) fractions (bottom) were tested for murine anti-Bet v 1 IgG. MW: Molecular weight marker; N: Native extract.

Supplementary Figure 6 Thiocyanate ELISA elution assay. IgE-binding of NEs and HMWFs PEs from Pp-N **(A)** and Bv-N **(B)** in the presence of KSCN at different molar concentrations. Representation of the results as KSCN concentration versus % reduction of IgE binding were used to calculate the avidity indexes.

Supplementary Figure 7 Mast cell activation test. Preliminary experiments were performed for all native extracts and HMWFs PEs, using two serum pools prepared with 5 individual sera from subjects sensitized to *P. pratense* and 5 to *B. verrucosa*. Results are displayed in contingency bar graphs for percentage of CD63+ or CD107a+ mast cells when stimulated with different concentrations of NEs and HMWFs PEs from Pp-N **(A)** and Bv-N **(B)**. The same analysis plus β-hexosaminidase activity was performed with individual sera using each stimulus at 10 μg/mL for native extract and PEs from Pp-N **(C to E)** and Bv-N **(F to H)**. When a statistically significant difference was found, the p-value is indicated at the top of the graphic. **p* < .05, ***p* < .01, ****p* < .001.

Supplementary Figure 8 Serum antibody response in mice immunized with NEs and P1 PEs. Specific IgG1, IgG2a and IgE in the serum of mice immunized with Pp-N and Pp-P1 (A to C) and Bv-N and Bv-P1 (E to G) measured by ELISA. The IgG2a/IgE ratio are also shown in (D) and (H), respectively. When a statistically significant difference was found, the p-value is indicated at the top of the graphic. **p* < .05, ***p* < .01, ****p* < .001, *****p* < .001.

## References

[1] Marsh DG Lichtenstein LM Campbell DH . Studies on “allergoids” prepared from naturally occurring allergens. I. Assay of allergenicity and antigenicity of formalinized rye group I component. Immunology. (1970) 18:705–22. PMC1455593 4192674

[2] Patterson R Suszko IM Zeiss CR Pruzansky JJ Bacal E . Comparison of immune reactivity to polyvalent monomeric and polymeric ragweed antigens. J Allergy Clin Immunol. (1978) 61:28–35. doi: 10.1016/0091-6749(78)90470-0, PMID: 618944

[3] Patterson R Suszko IM Bacal E Zeiss CR Kelly JF Pruzansky JJ . Reduced allergenicity of high molecular weight ragweed polymers. J Allergy Clin Immunol. (1979) 63:47–50. doi: 10.1016/0091-6749(79)90161-1, PMID: 102675

[4] Grammer LC Shaughnessy MA Suszko IM Shaughnessy JJ Patterson R . A double-blind histamine placebo-controlled trial of polymerized whole grass for immunotherapy of grass allergy. J Allergy Clin Immunol. (1983) 72:448–53. doi: 10.1016/0091-6749(83)90580-8, PMID: 6355247

[5] Grammer LC Shaughnessy MA Finkle SM Shaughnessy JJ Patterson R . Safety and immunogenicity of immunotherapy with polymerized tree, grass, and ragweed in patients with multiple inhalant sensitivities. J Allergy Clin Immunol. (1986) 77:53–8. doi: 10.1016/0091-6749(86)90322-2, PMID: 3944375

[6] Grammer LC Shaughnessy MA Suszko IM Shaughnessy JJ Patterson R . Persistence of efficacy after a brief course of polymerized ragweed allergen: a controlled study. J Allergy Clin Immunol. (1984) 73:484–9. doi: 10.1016/0091-6749(84)90359-2, PMID: 6707391

[7] Metzger WJ Patterson R Zeiss R Irons JS Pruzansky JJ Suszko IM . Comparison of polymerized and unpolymerized antigen E for immunotherapy of ragweed allergy. N Engl J Med. (1976) 295:1160–4. doi: 10.1056/NEJM197611182952103, PMID: 824555

[8] Kelly JF Zeiss CR Patterson R Levitz D Suszko IM . Polymerized whole ragweed: Human safety and immune response. J Allergy Clin Immunol. (1980) 65:50–6. doi: 10.1016/0091-6749(80)90176-1, PMID: 6153084

[9] Grammer LC Zeiss CR Suszko IM Shaughnessy MA Patterson R . A double-blind, placebo-controlled trial of polymerized whole ragweed for immunotherapy of ragweed allergy. J Allergy Clin Immunol. (1982) 69:494–9. doi: 10.1016/0091-6749(82)90173-7, PMID: 7042799

[10] Lozano J Cruz MJ Piquer M Giner MT Plaza AM . Assessing the efficacy of immunotherapy with a glutaraldehyde-modified house dust mite extract in children by monitoring changes in clinical parameters and inflammatory markers in exhaled breath. Int Arch Allergy Immunol. (2014) 165:140–7. doi: 10.1159/000368832, PMID: 25471080

[11] Ibarrola I Sanz ML Gamboa PM Mir A Benahmed D Ferrer A . Biological characterization of glutaraldehyde-modified Parietaria judaica pollen extracts. Clin Exp Allergy. (2004) 34:303–9. doi: 10.1111/j.1365-2222.2004.01859.x, PMID: 14987312

[12] Sola JP Pedreno Y Cerezo A Penalver-Mellado M . Development and characterization of an allergoid of cat dander for immunotherapy. Allergol Immunopathol (Madr). (2018) 46:491–8. doi: 10.1016/j.aller.2017.12.003, PMID: 29342409

[13] Guzman-Fulgencio M Caballero R Lara B Mena M Tejera M Sastre A . Safety of immunotherapy with glutaraldehyde modified allergen extracts in children and adults. Allergol Immunopathol (Madr). (2017) 45:198–207. doi: 10.1016/j.aller.2016.08.008, PMID: 27939406

[14] Patterson R Suszko IM McIntire FC . Polymerized ragweed antigen E. I. Preparation and immunologic studies. J Immunol. (1973) 110:1402–12. doi: 10.4049/jimmunol.110.5.1402, PMID: 4633300

[15] Patterson R Suszko IM Pruzansky JJ Zeiss CR . Polymerized ragweed antigen e. ii. *in vivo* elimination studies and reactivity with IgE antibody systems. J Immunol. (1973) 110:1413–8. doi: 10.4049/jimmunol.110.5.1413, PMID: 4121425

[16] Patterson R Suszko IM Pruzansky JJ Zeiss CR Metzger WJ Roberts M . Polymerization of mixtures of grass allergens. J Allergy Clin Immunol. (1977) 59:314–9. doi: 10.1016/0091-6749(77)90053-7, PMID: 66243

[17] Mistrello G Brenna O Roncarolo D Zanoni D Gentili M Falagiani P . Monomeric chemically modified allergens: immunologic and physicochemical characterization. Allergy. (1996) 51:8–15. doi: 10.1111/j.1398-9995.1996.tb00003.x, PMID: 8721522

[18] Zimmer J Bonertz A Vieths S . Quality requirements for allergen extracts and allergoids for allergen immunotherapy. Allergol Immunopathol (Madr). (2017) 45 Suppl 1:4–11. doi: 10.1016/j.aller.2017.09.002, PMID: 29128092

[19] Mugnaini G Gelli R Mori L Bonini M . How to cross-Llnk Gglatin: The effect of glutaraldehyde and glyceraldehyde on the hydrogel properties. ACS Appl Polym Mater. (2023) 5:11. doi: 10.1021/acsapm.3c01676, PMID: 41710754

[20] Ruijgrok JM de Wijn JR Boon ME . Glutaraldehyde crosslinking of collagen: effects of time, temperature, concentration and presoaking as measured by shrinkage temperature. Clin Mater. (1994) 17:23–7. doi: 10.1016/0267-6605(94)90044-2, PMID: 10150174

[21] Wen P Gao J Zhang Y Li X Long Y Wu X . Fabrication of chitosan scaffolds with tunable porous orientation structure for tissue engineering. J Biomater Sci Polym Ed. (2011) 22:19–40. doi: 10.1163/092050609X12572464984331, PMID: 20557692

[22] Cases B Ibanez MD Tudela JI Sanchez-Garcia S Del Rio PR Fernandez EA . Immunological cross-reactivity between olive and grass pollen: implication of major and minor allergens. World Allergy Organ J. (2014) 7:11. doi: 10.1186/1939-4551-7-11, PMID: 24940475 PMC4045862

[23] Sivill S Iborra S Cantillo JF . Efficient experimental method for purifying allergens from aqueous extracts. Methods. (2024) 229:63–70. doi: 10.1016/j.ymeth.2024.06.008, PMID: 38917960

[24] Ciordia S Santos FM Dias JML Lamas JR Paradela A Alvarez-Sola G . Refinement of paramagnetic bead-based digestion protocol for automatic sample preparation using an artificial neural network. Talanta. (2024) 274:125988. doi: 10.1016/j.talanta.2024.125988, PMID: 38569368

[25] Manzano AI Javier Canada F Cases B Sirvent S Soria I Palomares O . Structural studies of novel glycoconjugates from polymerized allergens (allergoids) and mannans as allergy vaccines. Glycoconj J. (2016) 33:93–101. doi: 10.1007/s10719-015-9640-4, PMID: 26603537 PMC4722057

[26] Groves P Rasmussen MO Molero MD Samain E Canada FJ Driguez H . Diffusion ordered spectroscopy as a complement to size exclusion chromatography in oligosaccharide analysis. Glycobiology. (2004) 14:451–6. doi: 10.1093/glycob/cwh037, PMID: 14693914

[27] El-Khouly F Lewis SA Pons L Burks AW Hourihane JO . IgG and IgE avidity characteristics of peanut allergic individuals. Pediatr Allergy Immunol. (2007) 18:607–13. doi: 10.1111/j.1399-3038.2007.00542.x, PMID: 18001431

[28] Kirshenbaum AS Akin C Wu Y Rottem M Goff JP Beaven MA . Characterization of novel stem cell factor responsive human mast cell lines LAD 1 and 2 established from a patient with mast cell sarcoma/leukemia; activation following aggregation of FcepsilonRI or FcgammaRI. Leuk Res. (2003) 27:677–82. doi: 10.1016/S0145-2126(02)00343-0, PMID: 12801524

[29] Lopez-Sanz C Sanchez-Martinez E Jimenez-Saiz R . Protocol to desensitize human and murine mast cells after polyclonal IgE sensitization. STAR Protoc. (2022) 3:101755. doi: 10.1016/j.xpro.2022.101755, PMID: 36223269 PMC9556792

[30] Fernandez-Gallego N Castillo-Gonzalez R Moreno-Serna L Garcia-Civico AJ Sanchez-Martinez E Lopez-Sanz C . Allergic inflammation triggers dyslipidemia via IgG signalling. Allergy. (2024) 79:2680–99. doi: 10.1111/all.16187, PMID: 38864116

[31] Soria I Lopez-Relano J Vinuela M Tudela JI Angelina A Benito-Villalvilla C . Oral myeloid cells uptake allergoids coupled to mannan driving Th1/Treg responses upon sublingual delivery in mice. Allergy. (2018) 73:875–84. doi: 10.1111/all.13396, PMID: 29319882 PMC5947296

[32] Zimmer J Vieths S Kaul S . Standardization and regulation of allergen products in the european union. Curr Allergy Asthma Rep. (2016) 16:21. doi: 10.1007/s11882-016-0599-4, PMID: 26874849

[33] Okuda K Urabe I Yamada Y Okada H . Reaction of glutaraldehyde with amino and thiol compounds. J Ferment Bioeng. (1991) 71:6. doi: 10.1016/0922-338X(91)90231-5

[34] Migneault I Dartiguenave C Bertrand MJ Waldron KC . Glutaraldehyde: behavior in aqueous solution, reaction with proteins, and application to enzyme crosslinking. Biotechniques. (2004) 37:790–6, 8-802. doi: 10.2144/04375RV01, PMID: 15560135

[35] Starchenka S Bell AJ Mwange J Skinner MA Heath MD . Molecular fingerprinting of complex grass allergoids: size assessments reveal new insights in epitope repertoires and functional capacities. World Allergy Organ J. (2017) 10:17. doi: 10.1186/s40413-017-0146-3, PMID: 28451054 PMC5402054

[36] Caraballo L Valenta R Puerta L Pomes A Zakzuk J Fernandez-Caldas E . The allergenic activity and clinical impact of individual IgE-antibody binding molecules from indoor allergen sources. World Allergy Organ J. (2020) 13:100118. doi: 10.1016/j.waojou.2020.100118, PMID: 32373267 PMC7195550

[37] Komlosi ZI Kovacs N Sokolowska M van de Veen W Akdis M Akdis CA . Highlights of novel vaccination strategies in allergen immunotherapy. Immunol Allergy Clin North Am. (2020) 40:15–24. doi: 10.1016/j.iac.2019.09.010, PMID: 31761116

[38] Lund L Henmar H Wurtzen PA Lund G Hjortskov N Larsen JN . Comparison of allergenicity and immunogenicity of an intact allergen vaccine and commercially available allergoid products for birch pollen immunotherapy. Clin Exp Allergy. (2007) 37:564–71. doi: 10.1111/j.1365-2222.2007.02687.x, PMID: 17430354

[39] Marsh DG Norman PS Roebber M Lichtenstein LM . Studies on allergoids from naturally occurring allergens. III. Preparation of ragweed pollen allergoids by aldehyde modification in two steps. J Allergy Clin Immunol. (1981) 68:449–59. doi: 10.1016/0091-6749(81)90199-8, PMID: 6171586

[40] Engeroff P Caviezel F Storni F Thoms F Vogel M Bachmann MF . Allergens displayed on virus-like particles are highly immunogenic but fail to activate human mast cells. Allergy. (2018) 73:341–9. doi: 10.1111/all.13268, PMID: 28787769

[41] Brazhnikov G Smolnikov E Litovkina A Jiang T Shatilov A Tulaeva I . Natural human Bet v 1-specific IgG antibodies recognize non-conformational epitopes whereas IgE reacts with conformational epitopes. Allergy. (2023) 78:3136–53. doi: 10.1111/all.15865, PMID: 37701941 PMC10952721

[42] Daeron M Malbec O Latour S Arock M Fridman WH . Regulation of high-affinity IgE receptor-mediated mast cell activation by murine low-affinity IgG receptors. J Clin Invest. (1995) 95:577–85. doi: 10.1172/JCI117701, PMID: 7860741 PMC295517

[43] Strait RT Morris SC Finkelman FD . IgG-blocking antibodies inhibit IgE-mediated anaphylaxis *in vivo* through both antigen interception and Fc gamma RIIb cross-linking. J Clin Invest. (2006) 116:833–41. doi: 10.1172/JCI25575, PMID: 16498503 PMC1378186

[44] Henmar H Lund G Lund L Petersen A Wurtzen PA . Allergenicity, immunogenicity and dose-relationship of three intact allergen vaccines and four allergoid vaccines for subcutaneous grass pollen immunotherapy. Clin Exp Immunol. (2008) 153:316–23. doi: 10.1111/j.1365-2249.2008.03710.x, PMID: 18647321 PMC2527365

[45] Calzada D Aranda T G MG Escutia MR Balsa D Alvarez J . Immunological mechanisms involved in the human response to a dog dander allergoid. Mol Immunol. (2022) 145:88–96. doi: 10.1016/j.molimm.2022.02.020, PMID: 35306358

[46] Rauber MM Wu HK Adams B Pickert J Bohle B Shamji MH . Birch pollen allergen-specific immunotherapy with glutaraldehyde-modified allergoid induces IL-10 secretion and protective antibody responses. Allergy. (2019) 74:1575–9. doi: 10.1111/all.13774, PMID: 30866053

