# Supplementary figures and images for: Impact of cross-linking stoichiometry on the structure and allergenicity of glutaraldehyde-polymerized allergen extracts
Source: Front Immunol. 2026 Feb 26;17:1748277. doi: 10.3389/fimmu.2026.1748277 (PMC12980651; doi:10.3389/fimmu.2026.1748277)

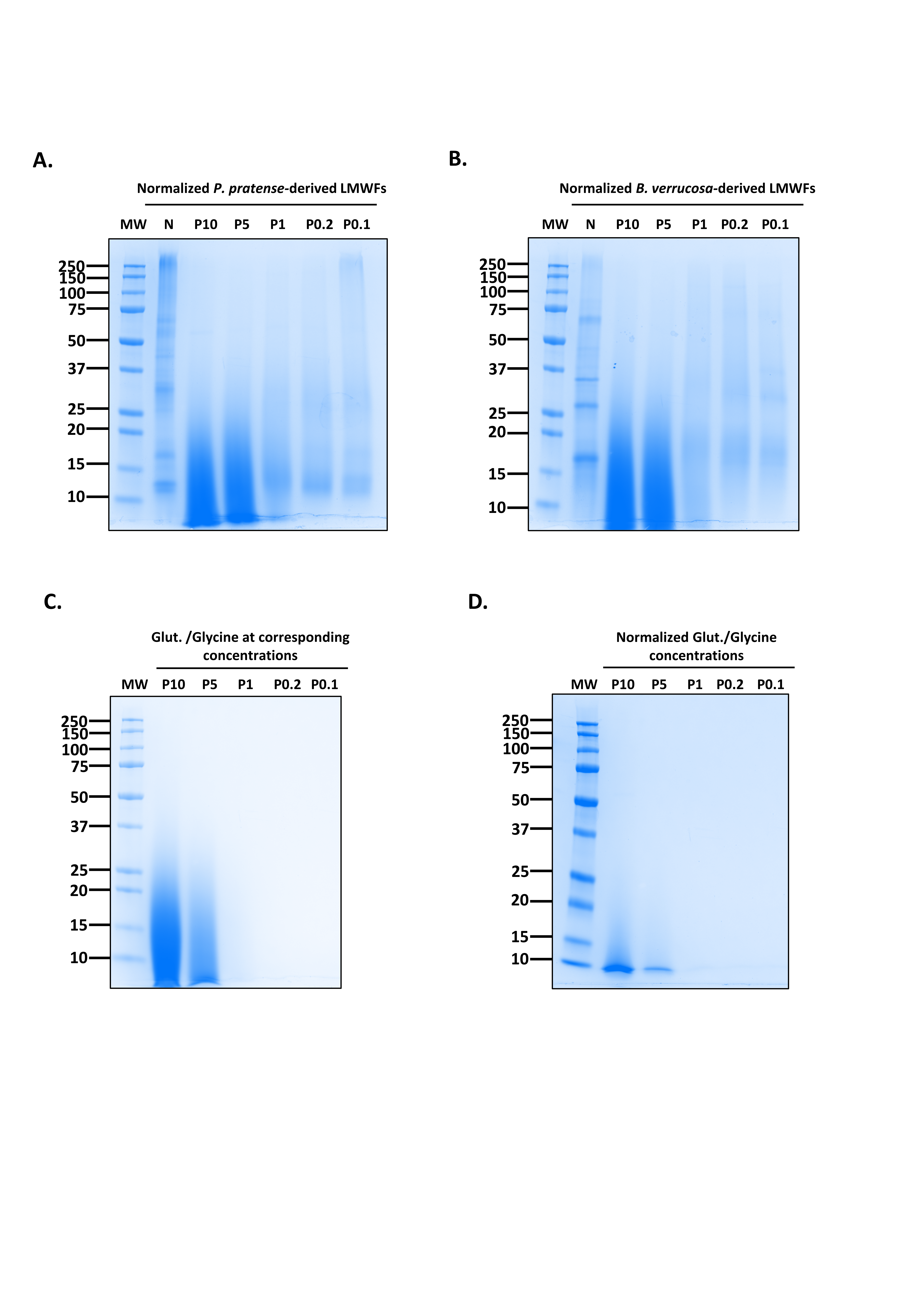

Supplement: Supplementary Figure 2 — Electrophoretic Analysis of LMWFs and bimolecular GA-Glycine Reaction products. Equal amounts of LMWFs (normalized) derived from the different polymerization of Pp-N (A) and Bv-N (B) were resolved by SDS-PAGE. To elucidate the nature of the <100 kDa intense smear that appeared in the LMWFs from P10 and P5 PEs, bimolecular GA–Glycine reaction resembling the conditions used for PEs preparation were resolved by SDS-PAGE, normalized by volume (C) or by the amount of GA-glycine (D), that were loaded in the wells. MW: Molecular weight marker; N: Native extract [file Image2.tif]

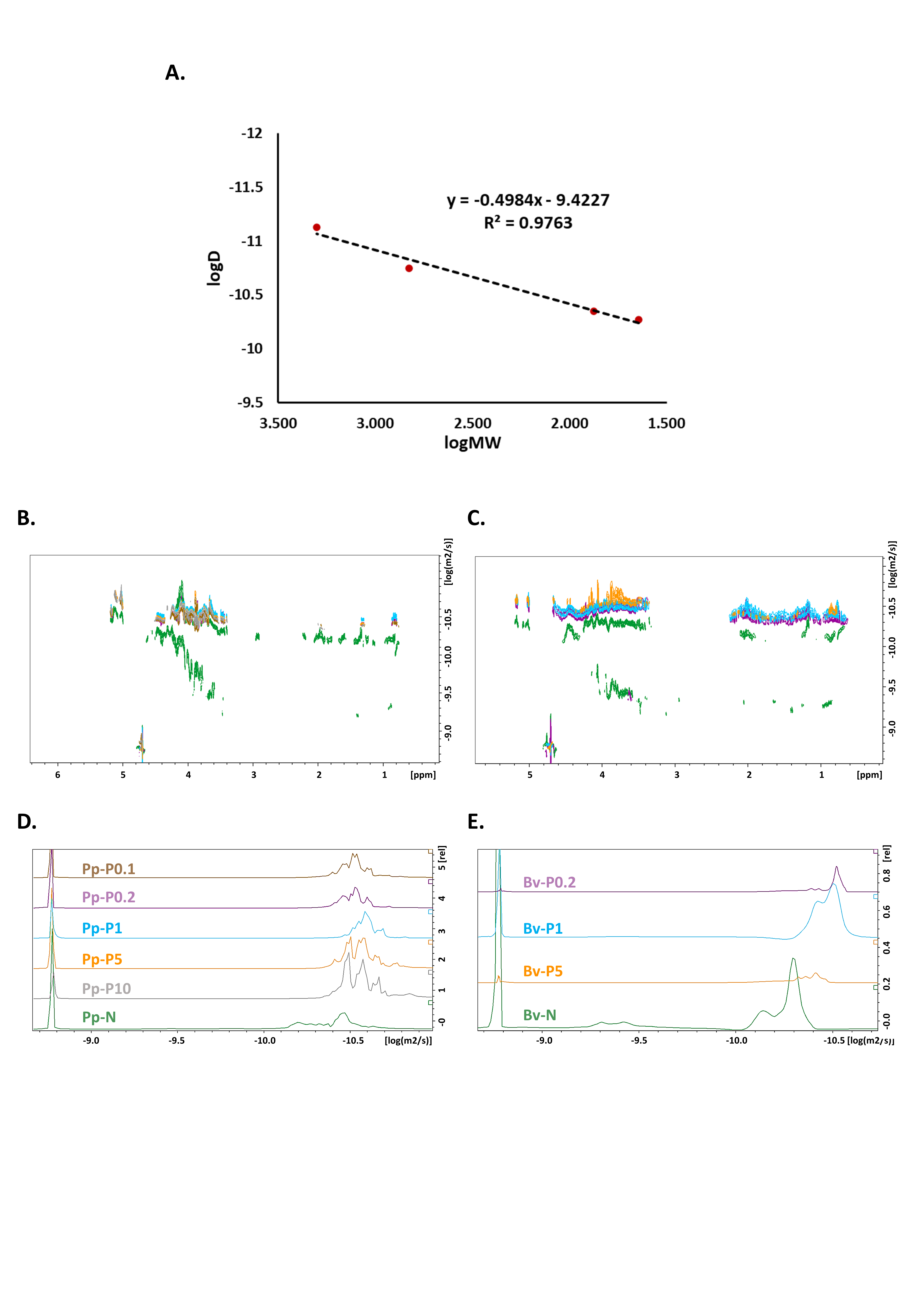

Supplement: Supplementary Figure 3 — Calibration curve and estimation of NEs and PEs molecular weights distribution by NMR data. (A) Plot of logD versus logMW for 4 globular proteins: ovalbumin (44 kDa), conalbumin (75 kDa), ferritin (669 kDa), and dextran (2,000 kDa). Superimposed 2D-DOSY spectra of Pp-N (B) and Bv-N (C) derived polymers. Colour codes are as follows: green for native extracts, gray for P10, orange for P5, blue for P1, magenta for P0.2, and brown for P0.1. (D and E) Column-wise sum projection of the DOSY spectra for the Pp-N and Bv-N derived polymers, respectively. [file Image3.tif]

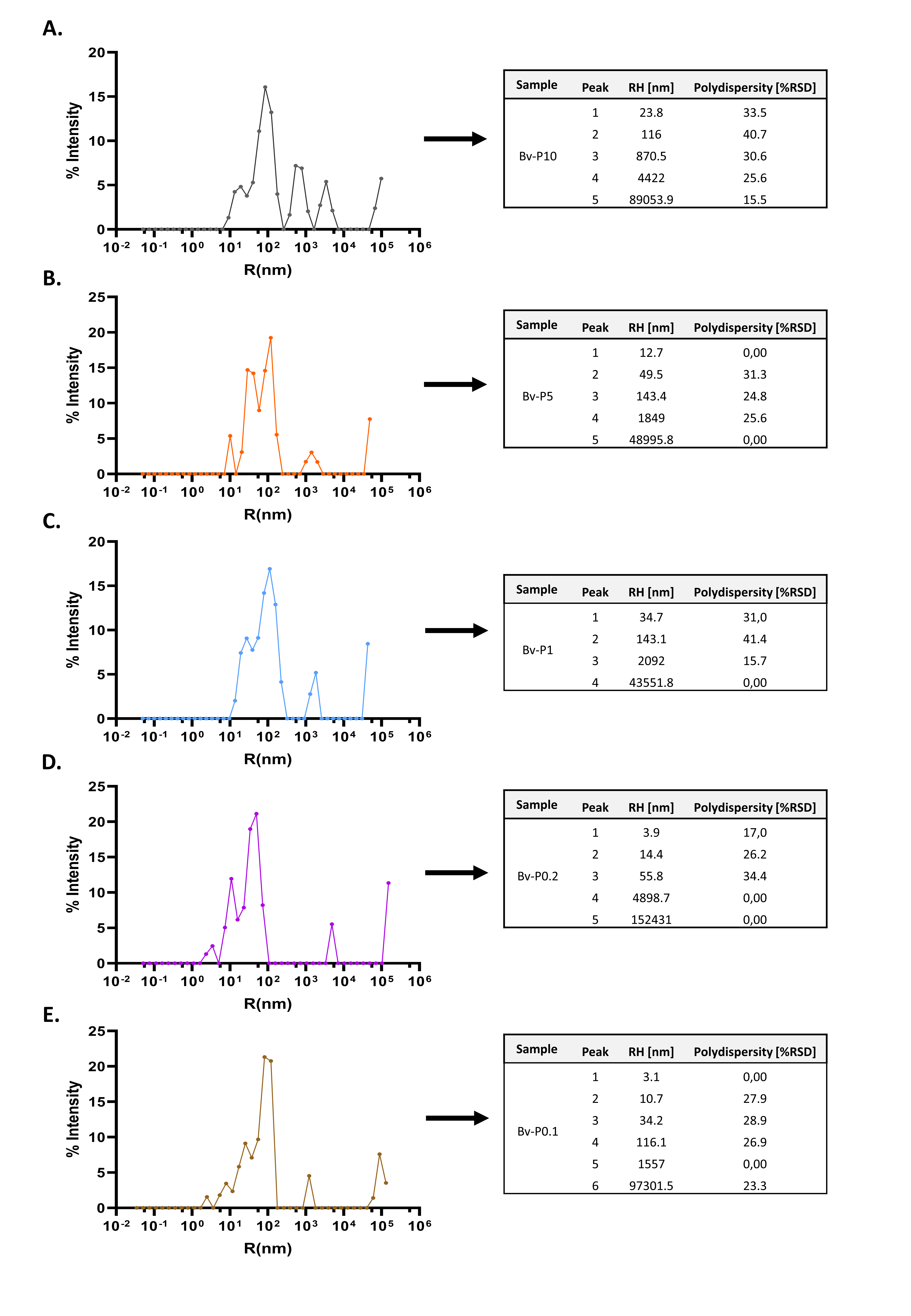

Supplement: Supplementary Figure 4 — Dynamic Light Scattering. Hydrodynamic radius vs Intensity distribution plots (right) and Intensity-weighted DLS peak parameters (left) are shown for Bv-P10 (A), Bv-P5 (B), Bv-P1 (C), Bv-P0.2 (D) and Bv-P0.1 (E). [file Image4.tif]

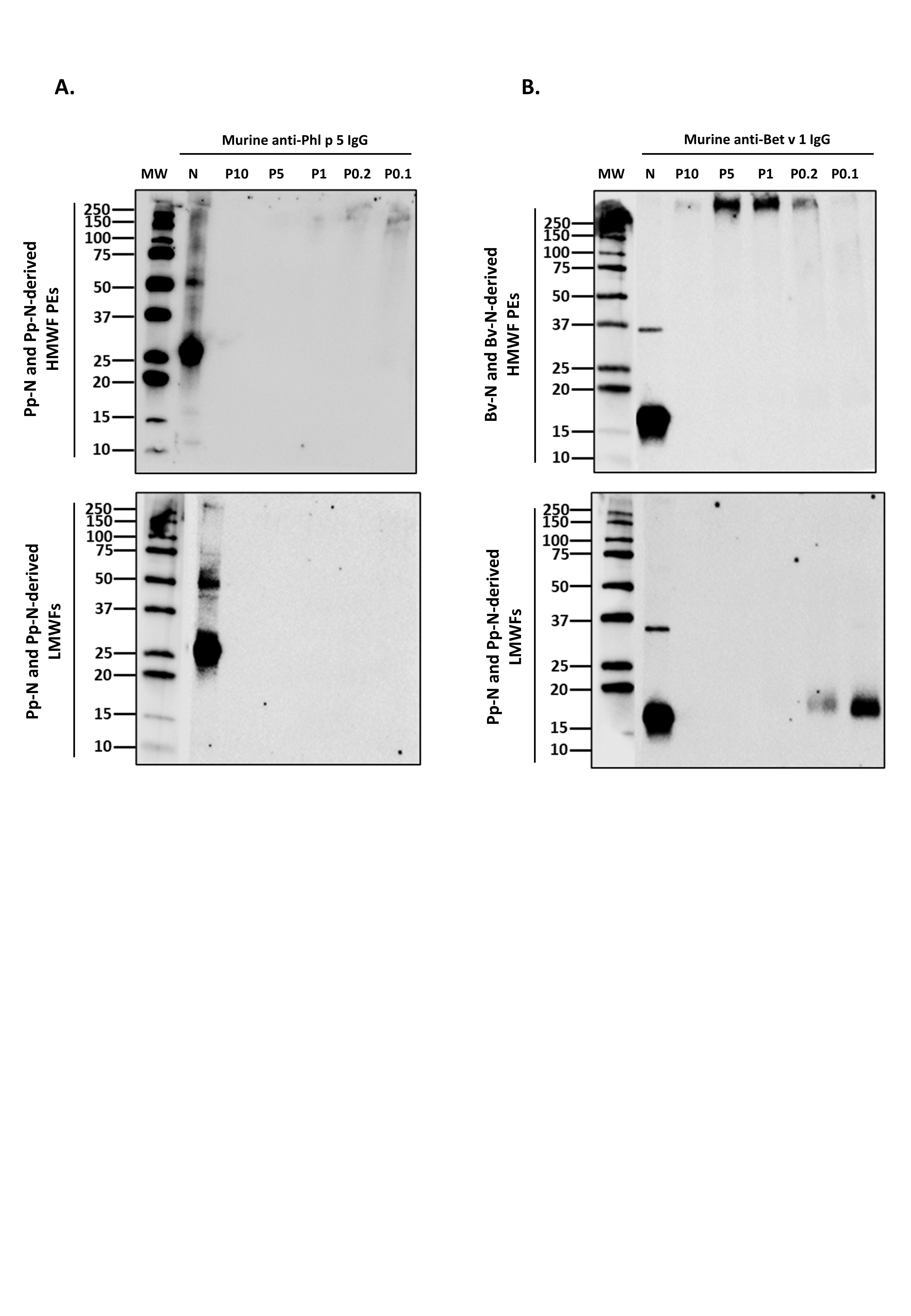

Supplement: Supplementary Figure 5 — Complementary WB analysis of NEs, HMWFs PEs, and LMWFs. (A) Pp-N and its derived polymers (top), and corresponding non-reactive fractions (bottom), were tested for murine anti-Phl p 5 IgG. (B) Bv-N and its derived polymers (top), and non-reactive (<100 kDa) fractions (bottom) were tested for murine anti-Bet v 1 IgG. MW: Molecular weight marker; N: Native extract. [file Image5.tif]

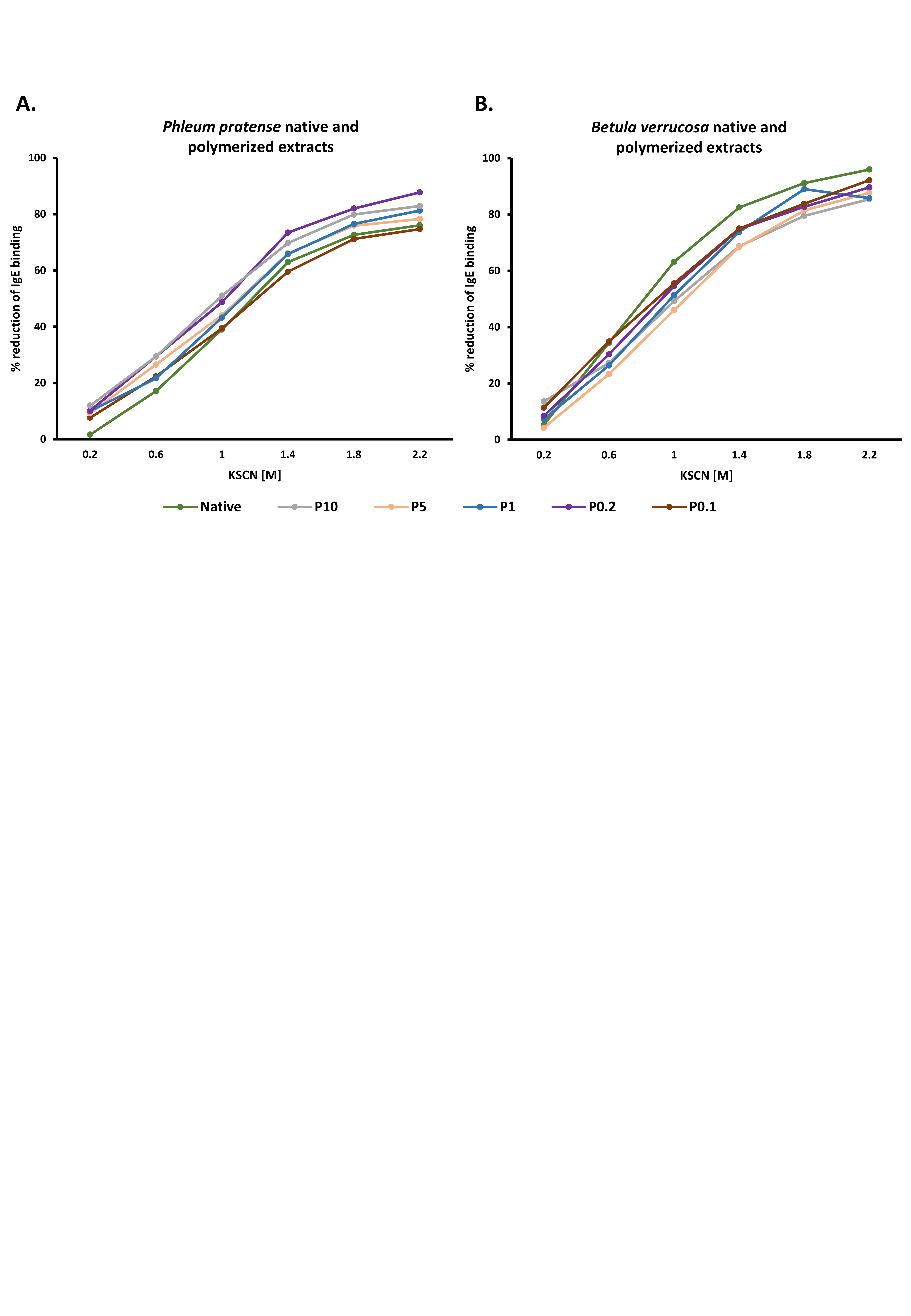

Supplement: Supplementary Figure 6 — Thiocyanate ELISA elution assay. IgE-binding of NEs and HMWFs PEs from Pp-N (A) and Bv-N (B) in the presence of KSCN at different molar concentrations. Representation of the results as KSCN concentration versus % reduction of IgE binding were used to calculate the avidity indexes. [file Image6.tif]

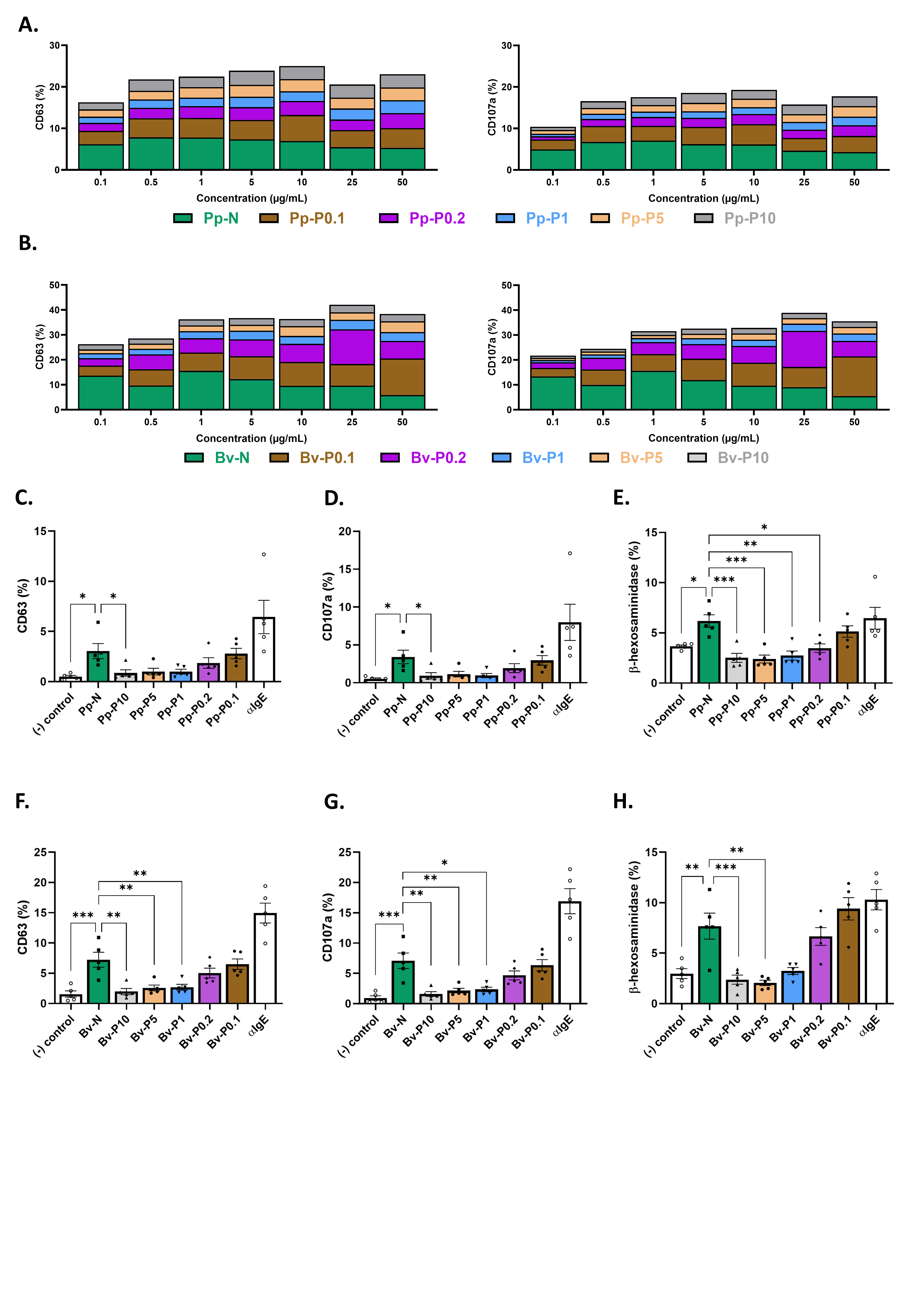

Supplement: Supplementary Figure 7 — Mast cell activation test. Preliminary experiments were performed for all native extracts and HMWFs PEs, using two serum pools prepared with 5 individual sera from subjects sensitized to P. pratense and 5 to B. verrucosa. Results are displayed in contingency bar graphs for percentage of CD63+ or CD107a+ mast cells when stimulated with different concentrations of NEs and HMWFs PEs from Pp-N (A) and Bv-N (B). The same analysis plus β-hexosaminidase activity was performed with individual sera using each stimulus at 10 μg/mL for native extract and PEs from Pp-N (C to E) and Bv-N (F to H). When a statistically significant difference was found, the p-value is indicated at the top of the graphic. *p < .05, **p < .01, ***p < .001. [file Image7.tif]

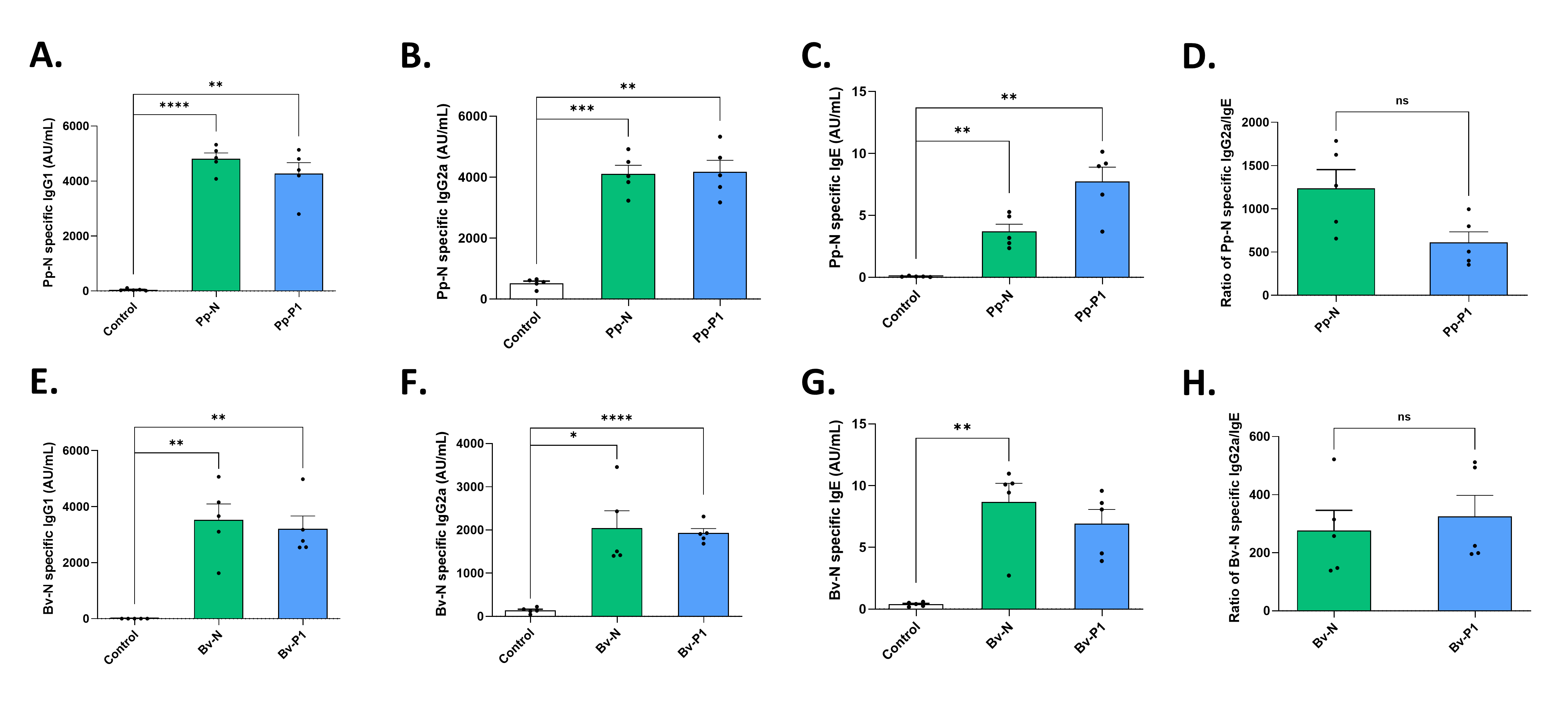

Supplement: Supplementary Figure 8 — Serum antibody response in mice immunized with NEs and P1 PEs. Specific IgG1, IgG2a and IgE in the serum of mice immunized with Pp-N and Pp-P1 (A to C) and Bv-N and Bv-P1 (E to G) measured by ELISA. The IgG2a/IgE ratio are also shown in (D) and (H), respectively. When a statistically significant difference was found, the p-value is indicated at the top of the graphic. *p < .05, **p < .01, ***p < .001, ****p < .001. [file Image8.tif]
