# Supplementary material for: Impact of cross-linking stoichiometry on the structure and allergenicity of glutaraldehyde-polymerized allergen extracts
Source: Front Immunol. 2026 Feb 26;17:1748277. doi: 10.3389/fimmu.2026.1748277 (PMC12980651; doi:10.3389/fimmu.2026.1748277)
Supplement: Supplementary file 9 [file Table1.docx]

**Supplementary Table 1.** Common proteins and allergens identified by mass spectrometry in the native (N) and polymerized (P) *Phleum pratense* (Pp) extract. Continuation of the Venn diagrams.

| **Common element** | **Number of common proteins** | **List of proteins** | | |
| --- | --- | --- | --- | --- |
| Pp-N  Pp-0.1  Pp-P1  Pp-P10 | 21 | **Pollen allergen Phl pI**  4-hydroxy-4-methyl-2-oxoglutarate aldolase  L-ascorbate oxidase  Transaldolase  Ferredoxin--NADP reductase, chloroplastic  Beta-glucosidase  5-methyltetrahydropteroyltriglutamate--Homocysteine S-methyltransferase  RNA helicase  Histone H4  Phosphoglucomutase (alpha-D-glucose-1,6-bisphosphate-dependent) | Thioredoxin reductase  Glucose-6-phosphate 1-epimerase  Glyceraldehyde-3-phosphate dehydrogenase  Malate dehydrogenase  Beta-galactosidase  NADH-cytochrome b5 reductase  Aminopeptidase  Protein-serine/threonine phosphatase  Aconitate hydratase  Glucose-6-phosphate isomerase  Ras-related protein RABA1f | |
| Pp-N  Pp-P1  Pp-P10 | 1 | Glycine-rich RNA-binding protein | | |
| Pp-N  Pp-0.1  Pp-P10 | 0 |  | | |
| Pp-N  Pp-0.1  Pp-P1 | 75 | Coatomer subunit gamma  Serine/threonine-protein phosphatase  5'-deoxynucleotidase  Pyridoxal 5'-phosphate synthase (glutamine hydrolyzing)  Adenylosuccinate synthetase, chloroplastic  DNA damage-inducible protein 1  Xaa-Pro aminopeptidase P  Spermidine synthase  UMP-CMP kinase  Dihydrolipoyl dehydrogenase  Glutamate dehydrogenase  Coproporphyrinogen oxidase  Cytosolic heat shock protein 90.2  Nucleoside diphosphate kinase  Proteasome subunit alpha type  Serine-threonine kinase receptor-associated protein ATP synthase subunit beta  Aldehyde dehydrogenase (NAD(+))  Guanosine nucleotide diphosphate dissociation inhibitor  Cysteine synthase  Succinate--CoA ligase [ADP-forming] subunit beta, mitochondrial  Glucose-1-phosphate adenylyltransferase  Peptide-methionine (S)-S-oxide reductase  LL-diaminopimelate aminotransferase  ATP citrate synthase  Carboxypeptidase  Adenylyl-sulfate kinase  Inositol-3-phosphate synthase  Thioredoxin-dependent peroxiredoxin  Glutathione S-transferase  Proteasome subunit beta  Phosphoenolpyruvate carboxylase  ATP-dependent 6-phosphofructokinase  Glucosidase II subunit alpha  6-phosphogluconate dehydrogenase, decarboxylating  Obg-like ATPase 1  3-deoxy-8-phosphooctulonate synthase | Isopentenyl-diphosphate  Delta-isomerase  UDP-arabinopyranose mutase  Acireductone dioxygenase  Arginase  Pyrophosphate--fructose 6-phosphate  1-phosphotransferase subunit beta  E2 ubiquitin-conjugating enzyme  Mitochondrial processing peptidase  Adenosine kinase  Leucine--tRNA ligase  3,4-dihydroxy-2-butanone kinase  Endoglucanase  Ubiquitin-NEDD8-like protein RUB1  TOM1-like protein 2  Inorganic diphosphatase  Heat shock 70 kDa protein, mitochondrial  Acyl-coenzyme A oxidase  Indole-3-glycerol-phosphate synthase  Alanine transaminase  Phosphomannomutase  Ubiquinone biosynthesis protein  Aspartate aminotransferase  E1 ubiquitin-activating enzyme  Peptidase M1 family protein  Metal-dependent protein hydrolase  Isocitrate dehydrogenase [NADP]  26S proteasome regulatory subunit RPN10  Ras-related protein Rab7  Pyridoxal kinase  ADP-ribosylation factor  Clathrin heavy chain  UDP-glucuronate decarboxylase Adenosylhomocysteinase  Phosphotransferase  UDP-glucose 6-dehydrogenase  Alpha-galactosidase  Purple acid phosphatase  2-oxoglutarate dehydrogenase, mitochondrial Ketol-acid reductoisomerase | |
| Pp-0.1  Pp-P1  Pp-P10 | 29 | **Pollen allergen Phl p 5.0101**  **Major pollen allergen Phl p 4**  **Pollen allergen Phl p 1**  **Pollen allergen Phl p 11**  **Pollen allergen Phl p 2**  **Phl p6 allergen**  **Polcalcin Phl p 7**  Anamorsin homolog dihydroorotase  Cell wall hydroxyproline-rich glycoprotein Glutathione dehydrogenase (ascorbate) Peptidylprolyl isomerase Phosphoribosylformylglycinamidine cyclo-ligase Small nuclear ribonucleoprotein-associated protein Actin | Pyrroline-5-carboxylate reductase  Fruit bromelain  RING-type E3 ubiquitin transferase  Ubiquitin-fold modifier-conjugating enzyme 1  Exopolygalacturonase  Pullulanase  Glucan endo-1,3-beta-D-glucosidase  Desiccation-related protein PCC13-62  Osmotin-like protein  Profilin-3  Choline kinase 2  Histone H2A  Oligopeptidase A  Thioredoxin | |
| Pp-N  Pp-P10 | 4 | Ras-related protein RABA2a  Heat shock protein 70  4-hydroxy-7-methoxy-3-oxo-3,4-dihydro-2H-1,4-benzoxazin-2-yl glucosidebeta-D-glucosidase  Phospholipase D | | |
| Pp-N  Pp-P1 | 12 | Importin subunit alpha  Transketolase  Nascent polypeptide-associated complex subunit beta  3-hydroxyisobutyryl-CoA hydrolase  S-(hydroxymethyl)glutathione dehydrogenase Pyruvate dehydrogenase E1 component subunit alpha | 3-isopropylmalate dehydrogenase Hydroxyacylglutathione hydrolase  Pre-mRNA-processing factor 19  Formate dehydrogenase, mitochondrial  Elongation factor 1-gamma  UTP-monosaccharide-1-phosphate uridylyltransferase | |
| Pp-N  Pp-0.1 | 10 | Glycerophosphodiester phosphodiesterase Glutathione transferase  Small ubiquitin-related modifier  Catalase  Elongation factor 1-alpha | CCT-beta  Pyruvate dehydrogenase E1 component subunit beta  Lactoylglutathione lyase  Methanethiol oxidase  Uridine 5'-monophosphate synthase | |
| Pp-P0.1  Pp-P10 | 1 | Calreticulin | | |
| Pp-P1  Pp-P10 | 2 | Phosphoglycerate mutase-like protein AT74H  Histone H3 | | |
| Pp-0.1  Pp-P1 | 165 | Ribonuclease  Hydroxyphenylpyruvate reductase  Pyruvate decarboxylase  Alpha-mannosidase  Stem-specific protein  Cytochrome c  Glycerol kinase  Small ribosomal subunit protein uS7c Argininosuccinate lyase  Ubiquitin receptor RAD23  Fructokinase  Methylenetetrahydrofolate reductase  Co-chaperone protein p23  Methyltransferase  Glutathione reductase  Fumarate hydratase  Oxoglutarate dehydrogenase (succinyl-transferring) 1,4-alpha-glucan branching enzyme  Proline--tRNA ligase  Xylose isomerase  Protein ROOT HAIR DEFECTIVE 3 homolog Putative gamma-glutamylcyclotransferase  Ribose-5-phosphate isomerase  Prefoldin subunit 1  Chalcone--flavanone isomerase  Pyruvate kinase  Golgi to ER traffic protein 4  Proline iminopeptidase  Dihydropyrimidine dehydrogenase (NADP(+)) Protein transport protein SEC23  Alpha-1,4 glucan phosphorylase  Vacuolar proton pump subunit B  Enoyl-[acyl-carrier-protein] reductase (NADH) Glutamate synthase (NADH)  Polyadenylate-binding protein  NAD(P)H-hydrate epimerase  Tryptophan--tRNA ligase  D-aminoacyl-tRNA deacylase  Alkaline/neutral invertase  Transcription factor Pur-alpha 1  Exocyst subunit Exo70 family protein  PHD finger protein ALFIN-LIKE  Coatomer subunit epsilon  ATP phosphoribosyltransferase  G3BP-like protein  Inositol-1,3,4-trisphosphate 5/6-kinase  Shikimate dehydrogenase (NADP(+))  Long-chain-fatty-acid--CoA ligase  Activator of 90 kDa heat shock protein ATPase Inositol-tetrakisphosphate 1-kinase  Beta-adaptin-like protein  Alpha-L-fucosidase  Histidinol-phosphatase  Ribokinase  Glutamate synthase (ferredoxin)  V-type proton ATPase subunit C  Beta-fructofuranosidase  Valine--tRNA ligase  Protein transport protein Sec24-like  Protein phosphatase methylesterase 1  Proliferating cell nuclear antigen Phosphoribosylglycinamide formyltransferase 1 Coatomer subunit beta Phosphoribosylaminoimidazolesuccinocarboxamide synthase  Asparagine--tRNA ligase  Arginine--tRNA ligase  Threonine synthase  Hsp17.2  Tubulin alpha chain  Phosphoserine aminotransferase  Alpha-N-acetylglucosaminidase  Glutamate decarboxylase  Guanine nucleotide-binding protein subunit beta-like protein A  NADH dehydrogenase [ubiquinone] 1 beta subcomplex subunit 9  Acyl-[acyl-carrier-protein] desaturase  Dihydroxy-acid dehydratase  Carbonic anhydrase  Myosin XI  (S)-2-hydroxy-acid oxidase  Heat shock protein 17.8  Assimilatory sulfite reductase (ferredoxin)  Glycine--tRNA ligase | mRNA cap-binding protein  D-3-phosphoglycerate dehydrogenase  Prolyl endopeptidase  Sucrose-phosphatase  Acetyltransferase  Component of pyruvate dehydrogenase complex  Plasma membrane ATPase  S-adenosylmethionine synthase  Serine hydroxymethyltransferase  Sucrose-phosphate synthase  Dihydrolipoyllysine-residue succinyltransferase Galactose oxidase  Aldose 1-epimerase  Vesicle-fusing ATPase  Aminomethyltransferase  4-alpha-glucanotransferase  Ubiquitin carboxyl-terminal hydrolase Methyltransferase PMT26  Tyrosine decarboxylase  Alpha-amylase  Methionine aminopeptidase  Protein disulfide-isomerase  Pectate lyase  4-coumarate--CoA ligase  Cyclic phosphodiesterase  Alanine--tRNA ligase  Protein MEMO1  Tryptophan synthase  Hypoxanthine phosphoribosyltransferase Glycylpeptide N-tetradecanoyltransferase  1-phosphatidylinositol 4-kinase  Phosphoserine phosphatase  Plant intracellular Ras-group-related LRR protein 3 Subtilisin-like protease SBT5.3  Non-specific serine/threonine protein kinase Coatomer subunit alpha  Isocitrate lyase  Anthranilate phosphoribosyltransferase  Uricase  3-hydroxyacyl-CoA dehydrogenase Diaminopimelate decarboxylase  Beta-ketoacyl-[acyl-carrier-protein] synthase III Ras-related protein RABC1  Adenylate kinase  Cell division cycle protein 48  Splicing factor U2af large subunit  Aspartate carbamoyltransferase Fumarylacetoacetase  Annexin  Glutamate--cysteine ligase  Cytochrome c oxidase subunit Vb  NEDD8-activating enzyme E1 regulatory subunit Sulfite oxidase  Phosphoacetylglucosamine mutase  Cinnamyl-alcohol dehydrogenase  Cytidine deaminase  Ferritin  Formate--tetrahydrofolate ligase  Protein-synthesizing GTPase  APOSTART  Succinate dehydrogenase [ubiquinone] flavoprotein subunit, mitochondrial  Hydroxymethylbilane synthase  SNARE-interacting protein KEULE  S-methyl-5-thioribose kinase  Xylulose kinase  S-formylglutathione hydrolase  Tripeptidyl-peptidase II  Ubiquitinyl hydrolase 1  Dipeptide epimerase  Glutaredoxin-dependent peroxiredoxin  Hsp organizing protein/stress-inducible protein Histidinol dehydrogenase  Alpha-soluble NSF attachment protein Pseudouridine-5'-phosphate glycosidase  Lysine--tRNA ligase  H(+)-transporting two-sector ATPase  Protein decapping 5  Glycosyltransferase  Methionine S-methyltransferase  Riboflavin kinase  Aspartate--tRNA ligase  40S ribosomal protein S26  GDSL esterase/lipase  Beta-amylase | |
| Pp-N | 42 | Polygalacturonase  Superoxide dismutase [Cu-Zn]  Ras-related protein RABA5a  Asparagine synthetase [glutamine-hydrolyzing]  Ras-related protein RABA3  Large ribosomal subunit protein bL9c  Eukaryotic translation initiation factor 3 subunit G Luminal-binding protein 5  RuBisCO large subunit-binding protein subunit alpha Subtilisin-like protease  Glutathione-specific gamma-glutamylcyclotransferase Ras-related protein RGP1  Oryzain alpha chain  Phosphoglycolate phosphatase  NAD(P)H dehydrogenase (quinone)  Maf-like protein  MOB kinase activator-like 1A  Multiple organellar RNA editing factor 8, chloroplastic/mitochondrial  Sulfate adenylyltransferase  Phospholipase C  Glutathione peroxidase | | 60S ribosomal protein L12  UTP--glucose-1-phosphate uridylyltransferase  Alpha-amylase  Cx9C motif-containing protein 4 Phosphoenolpyruvate carboxykinase (ATP) Monodehydroascorbate reductase (NADH)  Cellulase  Coatomer subunit beta  NADPH--cytochrome P450 reductase  L-ascorbate peroxidase  Alcohol dehydrogenase  Non-specific serine/threonine protein kinase Autophagy-related protein 18a  Pyrophosphate--fructose 6-phosphate 1-phosphotransferase subunit alpha  Adenylyl cyclase-associated protein Phosphoethanolamine N-methyltransferase Peroxidase  Probable 6-phosphogluconolactonase  Protein disulfide-isomerase  Stress up-regulated Nod 19  Phosphoglycerate kinase |
| Pp-P10 | 10 | **Group V allergen Phl p 5.0206**  IST1-like protein  Histone H3.3-like  Fructosyltransferase-like protein  Histone H3.2 | | Cytochrome P450 716B1  NAD-dependent epimerase/dehydratase  Dynein light chain  NADPH-dependent pterin aldehyde reductase Fructose-bisphosphate aldolase |
| Pp-P1 | 70 | 3-dehydroquinate synthase  Non-reducing end alpha-L-arabinofuranosidase Histone H2B  Glutamate--tRNA ligase  Diacylglycerol kinase  Pyridoxal 5'-phosphate synthase  Protein CHROMATIN REMODELING 4  Farnesyl pyrophosphate synthase  Nardilysin  Cytokinin dehydrogenase  Protein RCC2  Profilin-4  GTP-binding protein SAR1A  Succinate dehydrogenase subunit 5, mitochondrial Pectin acetylesterase  Transposase (Putative), gypsy type  Shikimate kinase family protein  Oryzain beta chain  1-(5-phosphoribosyl)-5-[(5-phosphoribosylamino)methylideneamino] imidazole-4-carboxamide isomerase, chloroplastic  Serine--tRNA ligase  Heparanase-like protein 3  Cytochrome b-c1 complex subunit Rieske, mitochondrial  Riboflavin synthase  Mitochondrial import inner membrane translocase subunit  Heat shock 70 kDa protein 17  Peptide-methionine (R)-S-oxide reductase  Fructan beta-(2,1)-fructosidase  Oxysterol-binding protein  T-complex protein 1 subunit gamma  Glutamine synthetase  Methionine--tRNA ligase  Eukaryotic translation initiation factor 3 subunit D UDP-N-acetylglucosamine diphosphorylase Potassium channel beta subunit | | Inosine triphosphate pyrophosphatase  Protein CutA, chloroplastic  VAMP-like protein YKT61  ATP synthase 24 kDa subunit, mitochondrial  30S ribosomal protein S3, chloroplastic  Mitogen-activated protein kinase  Pentatricopeptide repeat-containing protein Diphosphomevalonate decarboxylase  6,7-dimethyl-8-ribityllumazine synthase  SIT4 phosphatase-associated family protein Threonine--tRNA ligase  Tyrosine--tRNA ligase  Macrophage migration inhibitory factor  Acid phosphatase 1  Calnexin  Nucleosome assembly protein 1  Autophagy-related protein  Hydroxyisourate hydrolase  Alpha-aminoacylpeptide hydrolase  Lactoylglutathione lyase  UDP-N-acetylglucosamine pyrophosphorylase Hydroxymethylglutaryl-CoA synthase Phosphatidylinositol 4-phosphate 5-kinase  Lysosomal Pro-X carboxypeptidase  Glutamine--tRNA ligase  Signal recognition particle subunit SRP68  Serine carboxypeptidase S28 family protein  Switch 2  ARM repeat superfamily protein  Protein RAE1  Acetylornithine transaminase  Inositol-1-monophosphatase  Succinate-semialdehyde dehydrogenase  Protein FAR1-RELATED SEQUENCE  Small nuclear ribonucleoprotein Sm D3  NADH dehydrogenase [ubiquinone] flavoprotein 2, mitochondrial |
| Pp-0.1 | 56 | Delta-1-pyrroline-5-carboxylate synthase  5'-3' exoribonuclease  CCT-theta  COP9 signalosome complex subunit 4  Multiple inositol polyphosphate phosphatase 1 Trehalose 6-phosphate phosphatase  Argininosuccinate synthase  Ultraviolet-B receptor UVR8  Ribosomal protein L3  Acetolactate synthase  Heat shock protein 90  Imidazole glycerol phosphate synthase hisHF  GrpE protein homolog  N-acyl-aliphatic-L-amino acid amidohydrolase Calcium-binding EF hand family protein  Protein FAM91A1  Mitochondrial Rho GTPase  Exosome complex exonuclease RRP44 homolog A 60S ribosomal protein L34  Eukaryotic translation initiation factor 3 subunit K Tubulin beta chain  Coatomer subunit delta  26S proteasome non-ATPase regulatory subunit 2 homolog  Cytochrome b-c1 complex subunit 7  Homoserine dehydrogenase  ATP synthase subunit alpha  Ubiquitin thioesterase OTU  Carbamoyl-phosphate synthase (glutamine-hydrolyzing) | | Galactokinase  Cytochrome b  Vacuolar protein sorting 45  Cystathionine beta-lyase  SEC1 family transport protein SLY1  30S ribosomal protein S4, chloroplastic  Threonine dehydratase  Selenoprotein O  Pyrrolidone-carboxylate peptidase  Probable bifunctional methylthioribulose-1-phosphate dehydratase/enolase-phosphatase E1  ATP-dependent Clp protease proteolytic subunit Argonaute 1  Small nuclear ribonucleoprotein Sm D2 Mitochondrial proton/calcium exchanger protein SAPK3  Xyloglucan endotransglucosylase/hydrolase Ethanolamine kinase  T-complex protein 1 subunit eta  Glucose-6-phosphate 1-dehydrogenase  Acyl-CoA oxidase  Isoleucine--tRNA ligase  Superoxide dismutase copper chaperone Mitochondrial dicarboxylate/tricarboxylate transporter-DTC  Translin  Aspartyl aminopeptidase  Transcription initiation factor IIA subunit 2  Apyrase  Calcium homeostasis endoplasmic reticulum protein |
