# Supplementary material for: Impact of cross-linking stoichiometry on the structure and allergenicity of glutaraldehyde-polymerized allergen extracts
Source: Front Immunol. 2026 Feb 26;17:1748277. doi: 10.3389/fimmu.2026.1748277 (PMC12980651; doi:10.3389/fimmu.2026.1748277)
Supplement: Supplementary file 10 [file Table2.docx]

**Supplementary Table 2.** Common proteins and allergens identified by mass spectrometryin the native (N) and polymerized (P) *Betula verrucosa* (Bv) extract. Continuation of the Venn diagrams.

| **Common element** | **Number of common proteins** | **List of proteins** | | | |
| --- | --- | --- | --- | --- | --- |
| Bv-N  Bv-P0.1  Bv-P1  Bv-P10 | 36 | 4-hydroxy-4-methyl-2-oxoglutarate aldolase Glutathione dehydrogenase (ascorbate)  Nucleoside diphosphate kinase  Actin  Polyadenylate-binding protein  Phosphopyruvate hydratase  Peptide-methionine (S)-S-oxide reductase  3-hydroxyisobutyryl-CoA hydrolase  Glutathione S-transferase  Translationally-controlled tumor protein  Peptidyl-prolyl cis-trans isomerase  NAD(P)H dehydrogenase (quinone)  Ferredoxin--NADP reductase, chloroplastic  5-methyltetrahydropteroyltriglutamate--homocysteine S-methyltransferase  Phosphoserine aminotransferase  UTP--glucose-1-phosphate uridylyltransferase RNA helicase  Tubulin-specific chaperone A  Superoxide dismutase | Histone H4  Phosphoglucomutase (alpha-D-glucose-1,6-bisphosphate-dependent)  Endoglucanase  Monodehydroascorbate reductase (NADH) Phospholipase D  Protein disulfide-isomerase  Endoplasmic reticulum chaperone BiP  Glucose-6-phosphate 1-epimerase  Malate dehydrogenase  Beta-galactosidase  Phosphoglycerate mutase (2,3-diphosphoglycerate-independent)  Peroxidase  Probable 6-phosphogluconolactonase ubiquitinyl hydrolase 1  MEMO1 family protein  UTP-monosaccharide-1-phosphate uridylyltransferase  Phosphoglycerate kinase | | |
| Bv-N  Bv-P1  Bv-P10 | 3 | Glutathione reductase  Calcium-dependent protein kinase  Histone H3 | | | |
| Bv-N  Bv-P0.1  Bv-P10 | 0 |  | | | |
| Bv-N  Bv-P0.1  Bv-P1 | 58 | DNA damage-inducible protein 1  Fructokinase  Xaa-Pro aminopeptidase P  UDP-glucose 4-epimerase  Fumarate hydratase  Dihydrolipoyl dehydrogenase  Proline--tRNA ligase  Glutamate dehydrogenase  Citrate synthase  Alpha-1,4 glucan phosphorylase  Vacuolar proton pump subunit B  Guanosine nucleotide diphosphate dissociation inhibitor  Cysteine synthase  D-aminoacyl-tRNA deacylase  Eukaryotic translation initiation factor 3 subunit B ATP citrate synthase  Inositol-3-phosphate synthase  S-(hydroxymethyl)glutathione dehydrogenase Thioredoxin-dependent peroxiredoxin  Proteasome subunit beta  Succinate--CoA ligase [ADP-forming] subunit alpha, mitochondrial  Cell division cycle protein 48 homolog  6-phosphogluconate dehydrogenase, decarboxylating  Obg-like ATPase 1  Glutathione peroxidase  V-type proton ATPase catalytic subunit A Glutamate decarboxylase  UDP-arabinopyranose mutase  Ras-related protein Rab11C | Assimilatory sulfite reductase (ferredoxin)  T-complex protein 1 subunit zeta  Acetyltransferase component of pyruvate dehydrogenase complex  S-adenosylmethionine synthase  Adenosine kinase  Serine hydroxymethyltransferase  30S ribosomal protein S3, chloroplastic Aminomethyltransferase  4-alpha-glucanotransferase  Inorganic diphosphatase  Aminobutyraldehyde dehydrogenase  Alanine transaminase  Formate dehydrogenase, mitochondrial  Tryptophan synthase  Aspartate aminotransferase  E1 ubiquitin-activating enzyme  Isocitrate dehydrogenase [NADP]  Annexin  Pyrophosphate--fructose 6-phosphate 1-phosphotransferase subunit alpha  Adenylyl cyclase-associated protein  NADH-cytochrome b5 reductase  Fructose-bisphosphate aldolase  ADP-ribosylation factor  Adenosylhomocysteinase  UDP-glucose 6-dehydrogenase  Malic enzyme  Aconitate hydratase  Glucose-6-phosphate isomerase  2-oxoglutarate dehydrogenase, mitochondrial | | |
| Bv-P0.1  Bv-P1  Bv-P10 | 74 | **Phenylcoumaran benzylic ether reductase Betv6**  **Polcalcin Bet v 4**  Ribonuclease  Phospholipase  A-2-activating protein  Coatomer subunit gamma  Glycerophosphodiester phosphodiesterase Cytochrome c  Pectinesterase  Ubiquitin receptor RAD23  Olee1-like protein  Protein BOLA2  Cinnamoyl-CoA reductase 1  Putative gamma-glutamylcyclotransferase Peptidylprolyl isomerase  L-ascorbate oxidase  Proline iminopeptidase  Small nuclear ribonucleoprotein-associated protein Glucan endo-1,3-beta-D-glucosidase  Pectin acetylesterase  1 Sc-3 protein  Enolase  Birch protein  Rho GDP-dissociation inhibitor 1  10 kDa chaperonin  Carboxypeptidase  Nitrile-specifier protein 5  N-acyl-aliphatic-L-amino acid amidohydrolase 4CL10  1-Sc1 protein  Small ubiquitin-related modifier  Ubiquitin-fold modifier-conjugating enzyme 1 Pirin-like protein  Monocopper oxidase-like protein SKS1  Beta-glucosidase  Isopentenyl-diphosphate Delta-isomerase  Tic22-like family protein | Cyclase  Amine oxidase  Cysteine proteinase inhibitor  Adenine phosphoribosyltransferase  Pectin lyase-like superfamily protein isoform 1 LEA1  Rhamnogalacturonan endolyase  Calmodulin  Aldose 1-epimerase  Thiol protease aleurain-like  PII protein  Probable bifunctional methylthioribulose-1-phosphate dehydratase/enolase-phosphatase E1 Alpha-glucosidase  Neutral ceramidase  Indole-3-glycerol-phosphate synthase  Chorismate mutase  Alanine--tRNA ligase  Transmembrane protein 33 homolog  Profilin-2  Calcyclin-binding protein  Lysosomal Pro-X carboxypeptidase  Splicing factor U2af large subunit  Glutamine--tRNA ligase  Non-specific lipid-transfer protein  Beta-fructofuranosidase  Thaumatin-like protein  Triosephosphate isomerase, cytosolic  Eukaryotic translation initiation factor isoform 4E THL2  Protein-serine/threonine phosphatase Hydroxymethylbilane synthase  Protein-tyrosine-phosphatase  Glutaredoxin-dependent peroxiredoxin  Elongation factor 1-gamma  Beta-hexosaminidase  Alpha-galactosidase  Aspartyl aminopeptidase  Purple acid phosphatase | | |
| Bv-N  Bv-P10 | 1 | Lactoylglutathione lyase | | | |
| Bv-N  Bv-P1 | 5 | Serine/threonine-protein phosphatase  Histone H2B  Sucrose-phosphatase | Pyruvate dehydrogenase E1 component subunit beta  Ras-related protein Rab7 | | |
| Bv-N  Bv-P0.1 | 19 | 5'-deoxynucleotidase  Pyruvate decarboxylase  Spermidine synthase Phosphoribosylformylglycinamidine cyclo-ligase Proteasome subunit alpha type  ATP synthase subunit beta  Glucose-1-phosphate adenylyltransferase  Nascent polypeptide-associated complex subunit beta  Adenylyl-sulfate kinase | Glutamate synthase (ferredoxin)  Phosphoenolpyruvate carboxylase  Proliferating cell nuclear antigen  Prolyl endopeptidase  Pyruvate dehydrogenase E1 component subunit alpha  Thioredoxin reductase  Calnexin  Profilin  S-formylglutathione hydrolase  Ras-related protein RABA1f | | |
| Bv-P1  Bv-P10 | 10 | Imidazole glycerol phosphate synthase hisHF YbhB/YbcL family Raf kinase inhibitor-like protein  Stress-induced protein KIN2-like  Phosphatidate phosphatase  Calcium-transporting ATPase | Subtilisin-like protease SBT1.7  Trehalase  AT-hook motif nuclear-localized protein Glycosyltransferase  Chitinase | | |
| Bv-P0.1  Bv-P10 | 7 | **Major pollen allergen Bet v 1-A**  O-fucosyltransferase family protein  Non-reducing end alpha-L-arabinofuranosidase  Sm protein G | Germin-like protein  Cytidine deaminase  Uroporphyrinogen decarboxylase | | |
| Bv-P0.1  Bv-P1 | 88 | DNA damage-binding protein 1  Beta-glucanase  Cell wall hydroxyproline-rich glycoprotein Methylenetetrahydrofolate reductase  Pyridoxal 5'-phosphate synthase  Biotin carboxylase  1,4-alpha-glucan branching enzyme  CCT-theta  Asparagine synthetase [glutamine-hydrolyzing] Enoyl-ACP reductase  L-gulonolactone oxidase  Coproporphyrinogen oxidase  Argininosuccinate synthase  Serine-threonine kinase receptor-associated protein TatD related DNase  Importin subunit alpha  Mannan endo-1,4-beta-mannosidase  LanC-like protein GCL2  RuBisCO large subunit-binding protein subunit alpha  Alkaline/neutral invertase  Acetate--CoA ligase  Transcription factor Pur-alpha 1  Glutathione-specific gamma-glutamylcyclotransferase  LL-diaminopimelate aminotransferase  Delta-pyrroline-5-carboxylate dehydrogenase 2 Heparanase-like protein 3  Ubiquitin thioesterase  Alanine--glyoxylate transaminase  4-hydroxy-tetrahydrodipicolinate synthase  Maf-like protein  Valine--tRNA ligase  ATP-dependent 6-phosphofructokinase Glucosidase II subunit alpha  Phosphoribosylamine--glycine ligase  20 kDa chaperonin, chloroplastic  3-phosphoshikimate 1-carboxyvinyltransferase Asparagine--tRNA ligase  3-hydroxyacyl-[acyl-carrier-protein] dehydratase 3-deoxy-8-phosphooctulonate synthase  60S ribosomal protein L27  Glutamine synthetase  Cystathionine gamma-synthase  (S)-2-hydroxy-acid oxidase | Protein-disulfide reductase  Pyridoxal phosphate homeostasis protein  Auxin efflux carrier component  Leucine--tRNA ligase  Sucrose-phosphate synthase  3-isopropylmalate dehydrogenase Phosphoenolpyruvate carboxykinase (ATP) Tropinone reductase I  Allergen Cora1.08_Cav01g11530  Glycine cleavage system P protein  Acyl-coenzyme A oxidase  Methionine aminopeptidase  Glyceraldehyde-3-phosphate dehydrogenase Acetyl-CoA C-acyltransferase  Cyclic phosphodiesterase  Carbohydrate-binding-like fold protein  Factor independent urate hydroxylase  (3R)-hydroxymyristoyl-[acyl-carrier-protein] dehydratase  Ribosomal protein L34e  Phosphoserine phosphatase  NADPH--cytochrome P450 reductase Methylmalonate-semialdehyde dehydrogenase (CoA acylating)  Serine protease EDA2  Aspartate carbamoyltransferase  Molybdopterin biosynthesis protein CNX1  Indole-3-acetic acid-amido synthetase GH3.6 Lactoylglutathione lyase  Mitochondrial import receptor subunit TOM20 Ferritin  Phosphoethanolamine N-methyltransferase Oligopeptidase A  Clathrin heavy chain  S-methyl-5-thioribose kinase  UDP-glucuronate decarboxylase  Xylulose kinase  Tripeptidyl-peptidase II  Phosphotransferase  Sucrose synthase  Succinate-semialdehyde dehydrogenase  GDP-mannose 4,6-dehydratase  Methionine S-methyltransferase  Eukaryotic peptide chain release factor GTP-binding subunit  Methanethiol oxidase  Agmatine deiminase  Ketol-acid reductoisomerase | | |
| Bv-N | 49 | Formin-like protein  Acetohydroxy-acid reductoisomerase  Ras-related protein RABC2a-like  Mitochondrial import inner membrane translocase subunit Tim21  Protein ROOT HAIR DEFECTIVE 3 homolog Triosephosphate isomerase  Pyruvate kinase  Dihydropyrimidine dehydrogenase (NADP(+)) Protein transport protein SEC23  Ornithine carbamoyltransferase  Alcohol dehydrogenase  NSF attachment protein  Cysteine proteinase  Protein phosphatase methylesterase 1  Tubulin beta chain  Oxysterol-binding protein Phosphoribosylaminoimidazolesuccinocarboxamide synthase  Threonine synthase  Aminocyclopropanecarboxylate oxidase  Tubulin alpha chain  ADP/ATP translocase  Pyrophosphate--fructose 6-phosphate 1-phosphotransferase subunit beta  Eukaryotic translation initiation factor 3 subunit D  Ubiquitin thioesterase OTU | mRNA cap-binding protein  RAB18  Plasma membrane ATPase  Mitogen-activated protein kinase  Elongation factor 1-alpha  Multifunctional fusion protein  Mevalonate kinase  Cytochrome c oxidase subunit  Ubiquitin carboxyl-terminal hydrolase  Tyrosine decarboxylase  Cinnamoyl-CoA reductase  Profilin-6  Glycylpeptide N-tetradecanoyltransferase  Non-specific serine/threonine protein kinase Coatomer subunit alpha  Hydroxymethylglutaryl-CoA synthase  Adenylate kinase  40S ribosomal protein S12  Histone H2A  Protein-synthesizing GTPase  Succinate dehydrogenase [ubiquinone] flavoprotein subunit, mitochondrial  Pre-mRNA cleavage factor Im 25 kDa subunit Quinol--cytochrome-c reductase  Beta-amylase  Lon protease homolog, mitochondrial | | |
| Bv-P10 | 7 | Alpha-mannosidase  Cytokinin dehydrogenase  Alpha-L-fucosidase  Peptide-methionine (R)-S-oxide reductase | | Peptidyl-tRNA hydrolase  Elongation factor Tu  Cor a 1.0601 | |
| Bv-P1 | 34 | **Major pollen allergen Bet v 1-C**  Co-chaperone protein p23  Thiolase II  Dihydrolipoamide acetyltransferase component of pyruvate dehydrogenase complex  40S ribosomal protein S4  Replication protein A subunit  2-hydroxyacyl-CoA lyase  Sulfurtransferase  CXXC motif containing zinc binding protein  RNA ligase/cyclic nucleotide phosphodiesterase Serine--tRNA ligase  Beta-adaptin-like protein  Geranylgeranyl transferase type-2 subunit alpha Urease  26S proteasome non-ATPase regulatory subunit 2 homolog  Sulfate adenylyltransferase  Prefoldin subunit 3 | | |  |
| Bv-P0.1 | 71 | B-like cyclin  40S ribosomal protein S24  Geranylgeranyl transferase type-2 subunit beta Glycerol kinase  Acylaminoacyl-peptidase  Ribosomal protein L14b/L23e  Farnesyl pyrophosphate synthase  Ras-related protein RABA5a  2-oxoadipate dioxygenase/decarboxylase  Ras-related protein RABA3  Protein phosphatase 1 regulatory subunit 7  Delta-aminolevulinic acid dehydratase  Aldehyde dehydrogenase (NAD(+))  Fructose-1,6-bisphosphatase, cytosolic  Xaa-Pro dipeptidase  Oleosin Transmembrane 9 superfamily member Ribosomal protein L3  Eukaryotic translation initiation factor 5A  DNA ligase  Pyrroline-5-carboxylate reductase  Aspartokinase  Subtilisin-like protease SBT4.14  Aspartate-semialdehyde dehydrogenase  Ras-related protein RABA2a  Inositol-tetrakisphosphate 1-kinase  2,4-dienoyl-CoA reductase [(3E)-enoyl-CoA-producing]  Phosphoglycolate phosphatase  Heat shock 70 kDa protein 17  NADH dehydrogenase [ubiquinone] 1 alpha subcomplex subunit 12  Aspartate kinase  Multiple organellar RNA editing factor 8, chloroplastic/mitochondrial  Farnesyl diphosphate synthase  Peptide deformylase  Nicotinate phosphoribosyltransferase | | | Carbonic anhydrase  Protein CutA, chloroplastic  ATP-dependent (S)-NAD(P)H-hydrate dehydratase  E2 ubiquitin-conjugating enzyme  Defective in cullin neddylation protein Dihydrolipoyllysine-residue succinyltransferase Glucosamine 6-phosphate N-acetyltransferase Glutamate--cysteine ligase, chloroplastic  Histone-lysine N-methyltransferase  Condensin-2 complex subunit H2  Cystathionine beta-lyase  6,7-dimethyl-8-ribityllumazine synthase Hydroxyacylglutathione hydrolase  Dynamin GTPase  26S proteasome non-ATPase regulatory subunit 1 homolog  4-coumarate--CoA ligase  Ras-related protein Rab11A Phosphatidylethanolamine N-methyltransferase Dihydroorotate dehydrogenase (quinone), mitochondrial  Autophagy-related protein  Xyloglucan endotransglucosylase/hydrolase  4-hydroxyphenylpyruvate dioxygenase  S-protein homolog  Alcohol dehydrogenase  H(+)-exporting diphosphatase  (DL)-glycerol-3-phosphatase 2  Pyridoxal kinase  mannose-6-phosphate isomerase  Inositol oxygenase  Thiamine thiazole synthase, chloroplastic  Profilin-5  Histidinol dehydrogenase  3-oxoacyl-[acyl-carrier-protein] reductase  Aspartate--tRNA ligase  FRIGIDA-like protein  Small nuclear ribonucleoprotein Sm D3 |
